# Psychosocial interventions for depression delivered by non-mental health specialists to people living with HIV/AIDS in low- and middle-income countries: A systematic review

**DOI:** 10.7189/jogh.12.04049

**Published:** 2022-06-11

**Authors:** Mia Du Zeying, Thulani Ashcroft, Durga Kulkarni, Vilas Sawrikar, Caroline A Jackson

**Affiliations:** 1 Usher Institute, University of Edinburgh, Teviot Place, Edinburgh, Scotland; 2 Department of Clinical and Health Psychology, School of Health in Social Sciences, University of Edinburgh, Teviot Place, Edinburgh, Scotland

## Abstract

**Background:**

Depression commonly co-exists with human immunodeficiency virus (HIV), but in low- and middle-income countries (LMICs), where the HIV burden is greatest, mental health resources are limited. These settings may benefit from psychosocial interventions delivered to people living with HIV/AIDS (PLWH) by non-mental health specialists. We aimed to systematically review randomised controlled trials (RCTs) that investigated the effectiveness of psychosocial interventions delivered by non-mental health specialists to prevent depression in PLWH in LMICs.

**Methods:**

We used a comprehensive electronic search strategy to identify RCTs of any stage, including pilot studies, which reported on the effectiveness of a psychosocial intervention on depression among adults living with HIV/AIDS in a LMIC setting. Screening, study selection and data extraction was completed independently by two authors. We assessed risk of bias using the Cochrane risk of bias (RoB) tool and performed a narrative synthesis.

**Results:**

We identified 3431 studies, from which we included 15 studies corresponding to 14 RCTs and a total of 3997 PLWH. Eleven studies were parallel RCTs, one was a stepped-wedged RCT, one was a full factorial RCT, one was a three-arm RCT and four were pilot studies. Studies were generally small, with eight including depression as a primary outcome. All but four trials included men and women and most studies followed participants for less than one year. Twelve trials had at least one domain in which there was a high risk of bias, with the remaining two trials having at least one domain of concern, due to lack of reporting of items. In 12 studies people in the intervention arm had statistically significantly (*P* < 0.05) lower or more reduced depressive symptom scores, or were less likely to have major depression, at final follow-up than people in the control group.

**Conclusions:**

Psychosocial interventions delivered by non-specialist mental health workers may be effective in preventing or reducing depression in PLWH in LMICs. However, existing studies are small with a relatively short follow-up period and have methodological limitations. Future trials should address these shortcomings, establish whether intervention effects are clinically meaningful and investigate cost-effectiveness.

An estimated 38 million people live with HIV [1], which among people aged 25-49 is the second leading cause of disability adjusted life years (DALYs) globally [2]. LMICs bear the brunt of the HIV morbidity and mortality burden, accounting for 98.7% of global DALYs and deaths due to HIV [3]. People living with HIV/AIDS (PLWH) in LMICs have two to three times the risk of developing depression compared to the general population [4]. Untreated and under-recognized mental illnesses, particularly depression, have a negative impact across the HIV care continuum from HIV prevention, diagnosis, enrolment in antiretroviral therapy (ART), to retention in HIV care [5]. It is therefore imperative to provide access to health care reducing the occurrence of depression among PLWH, particularly in LMICs. To that end, research suggests that psychosocial interventions, such as social support, counselling, psychoeducation, and psychological therapy help alleviate the impacts of depression among individuals living with HIV [6]. Psychosocial interventions target psychosocial risk factors associated with emotional distress to either prevent the onset of depression, ameliorate clinical depression, or promote recovery by improving patient’s well-being and quality of life [6].

Previous systematic reviews have examined the effectiveness of psychosocial interventions on improving mental health in general in PLWH, two of which focused solely on studies from LMICs [7,8] with the others largely including studies from high income countries (HICs) [6,9]. Randomised controlled trials (RCTs) included in these reviews primarily focused on cognitive behaviour therapies (CBT), mostly delivered by mental health specialists [6,7,9], with the fourth review including pharmaceutical interventions [8]. Overall, CBT interventions moderately improved mental health in people with HIV, including in LMIC settings. However, standard delivery of these approaches (ie, via mental health specialists) may not be cost-effective for countries with limited specialist mental health resources [10]. There is increasing evidence that delegating tasks of mental illness detection and management to non-specialist health workers such as nurses, general practitioners and community workers (known as “task shifting”) could alleviate the mental health burden in various patient groups in LMICs [11]. This task shifting approach addresses issues arising from competing health demands, financial constraints, low mental health literacy, high levels of stigma and shortage of skilled mental health workers in these settings [12,13]. Consequently, studies have investigated the effectiveness of psychosocial interventions (including social support, counselling, psychoeducation, and psychological therapy) delivered by non-specialist mental health workers, such as nurses and community workers, on depression in PLWH. The aforementioned reviews of studies in LMICs did not specifically investigate the effectiveness of psychosocial interventions delivered by non-specialist mental health workers in PLWH, with just five of the 31 primary studies included across both reviews [7,8] adopting this approach. Additional studies have been published since these reviews were conducted. Study findings suggest that this approach may be effective in reducing depression PLWH [14,15], although not all studies report such an effect [16].

Therefore, whilst there is emerging evidence that psychosocial interventions may improve mental well-being in general in PLWH in LMICs, the effectiveness and methodological quality of such interventions delivered by non-specialist mental health workers is unclear and yet to be systematically reviewed. Thus, we aimed to systematically identify, critically appraise and synthesise RCTs that investigated the effectiveness of non-specialist mental health delivered psychosocial interventions to reduce depression in PLWH in LMICs.

## METHODS

### Search strategy

This systematic review is reported in line with the PRISMA statement [17]. We designed an extensive and comprehensive electronic search strategy which included medical subject headings and free-text words for the following: HIV and AIDS; psychosocial interventions; geographical locations; RCTs; mental health and depression (Appendix S1 in the **Online Supplementary Document**). We adapted search terms for psychosocial interventions from previous related reviews [10,18,19]. We searched Medline, Embase, PsycInfo, Web of Knowledge, Cochrane library, Global health and Global index medicus from point of inception to August 31, 2021 inclusive, without setting any language restrictions. Two authors (ZD and TA or DK) independently screened all titles and abstracts and the full-text of all potentially relevant articles, with disagreements resolved through discussion with a fourth reviewer (CAJ). We also searched unpublished or ongoing studies in ClinicalTrials.gov.

### Study selection

We used the Population, Intervention, Comparison and Outcome (PICO) framework when devising the systematic review question as follows: in PLWH (population) what is the effect of psychosocial interventions delivered by non-mental health specialists (intervention) compared to standard or other care (comparison) on depression (outcome)? We included both pilot and full-size RCTs which: included participants aged 18 years or over; evaluated effectiveness of psychosocial interventions targeting social or psychological aspects at individual, family, community or social levels; reported on depression status at both baseline and post-intervention; and were carried out in LMICs as defined by the World Bank. We excluded: perinatal studies and caregiver-child dyad studies where the focus was on improving mental health or cognitive performance in children or pregnant women; and studies where the deliverers had certificates or degrees in psychology such as psychiatrist, psychologists, mental health nurses or experienced mental health counsellors.

### Data extraction and analysis

Data items were independently extracted by two authors (ZD and TA or DK), with disagreements or uncertainties resolved after discussion, including with a third author where necessary (CAJ). We used a standardised data extraction form to extract information on the following data items: study characteristics; participant characteristics; intervention characteristics; details of comparison groups and retention rates in each group; depression ascertainment and measurement; and study results. We also recorded whether studies followed the principle of intention-to-treat. Since included studies were heterogeneous in terms of population and intervention characteristics, we performed a narrative synthesis rather than a meta-analysis.

### Assessment of risk of bias

We (ZD and TA or DK) used the Cochrane risk of bias (RoB) assessment tool [20] to assess risk of bias in each study. This consists of seven domains (randomised sequence generation, allocation concealment, blinding of participants and personnel, blinding of outcome assessment, incomplete outcome data, selecting reporting and other bias). For each domain, we directly cited evidence from studies or their protocols to support judgement of assessment and rated each domain as low or high risk of bias or some concerns. Where there were missing data or uncertainties based on the information given in articles we contacted authors of studies for more information before assessing the domain as ‘some concerns’. When assessing for ‘other bias’ we considered the following: balance of baseline characteristics; participant receipt of concurrent treatments such as other psychological therapies or pharmaceutical treatments; contamination of intervention between groups were collected to support the judgement; small sample size; and (with respect to cluster RCTs), loss of clusters, unit of analysis and adjustment for clustering. We constructed a ‘risk of bias’ graph and summary figures using the robvis package in RStudio (Version 4.0.1) (RStudio, Inc, Boston, USA). This review is reported in accordance with the PRISMA guidelines [17].

## RESULTS

### Study selection

**Figure 1:**
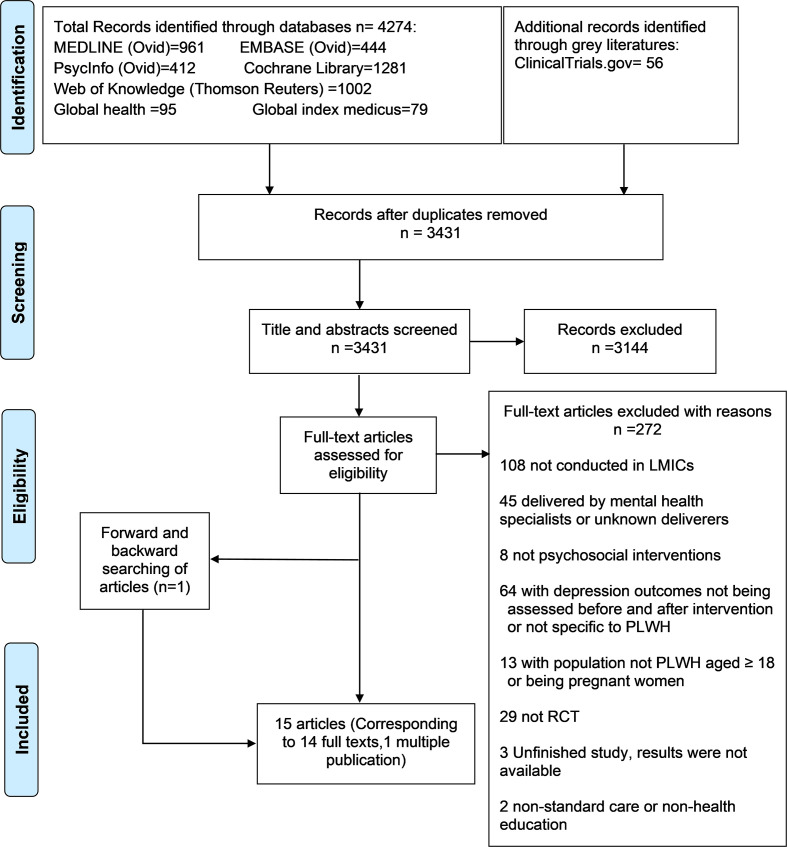
Flowchart of study selection.

Our search strategy identified 3431 studies after de-duplication. Following screening of titles and abstracts, we excluded 3144 studies that did not meet the eligibility criteria (**Figure 1**). We reviewed the full text of 287 potentially eligible studies and excluded 272 studies (Appendix S2 in the **Online Supplementary Document**). We ultimately included 15 studies [14-16,21-32] which corresponded to 14 RCTs.

### Study characteristics

Characteristics of the 14 RCTs are described in **Tables 1** and 2 and summarised here. Four trials were pilot studies [21,23,27,28]. There were eleven parallel RCTs [14-16,21,26-32], one stepped-wedge RCT [22], one full factorial RCT [23], one three-arm RCT [25]. Six studies were conducted in China [14,21,25,26,30,31], two in South Africa [16,28], one in Tanzania [22], one in Argentina [23], one in Uganda [15], one in India [27] and two in Kenya [29,32]. Eleven trials were conducted in urban areas or a mixture of urban and rural [14,16,21-23,25,28-32], whilst three [15,26,27] were carried out in rural areas. Participants were mainly recruited by health providers or research staffs in HIV clinics or health clinic centres, except for in one study, where they were recruited via social media or posters in public areas by peer fieldworkers [25]. The study population size ranged from 41 [21] to 1140 [15] people and a total of 3997 participants were recruited across all 14 studies. Three trials [21,27,32] included only women with HIV/AIDS, one trial only recruited HIV-infected men who have sex with men [25], and the remaining trials recruited both women and men. Nine trials [16,21-23,26,27,29-31] did not specify any eligibility criteria relating to depression status at baseline. Of the remaining five trials, one included PLWH with depression scores ≥16 assessed by Centre for Epidemiological Studies-Depression (CES-D) scale [14], two included PLWH with major depression [32] or mild to moderate depression [15] defined by Mini International Neuropsychiatric Interview depression module (MINI), and one with major depression defined by Structural Clinical Interview for a DSM IV Diagnosis [28]. One trial excluded participants with severe depression at baseline, as measured by the Patient Health Questionnaire-9 scale [25]. Depression was included as a primary outcome in 8 studies [14,15,21,25-28,32] and as a secondary outcome in 6 studies [16,22,23,29-31]. One trial used the MINI to assess diagnosis of major depression [15] (**Table 3**). The remaining measured depressive symptoms, using standardised depression screening or assessment scales, most commonly the CES-D scale.

**Table 1:** Characteristics of studies included in the systematic review

First author (year)	Country (urban/rural setting)	Study design*	Trial participants	Sample size, Intervention | control	Female (%)	Mean age, years (SD).† Intervention | control	Retention (%), Intervention | control	ITT
Chen (2018) [21]	China (urban)	Pilot parallel RCT	Women aged ≥18 with HIV/AIDS	21 | 20	100	40.6 (11.2) | 43.1 (10.2)	100 | 90	YES
Fawzi (2019) [22]	Tanzania (urban)	Stepped-wedge RCT	PLWH aged ≥18; currently on ART (≥3 mo); being able to invite 10 members vulnerable to infecting HIV	All: 458	57.0	Men: <40: 77% |≥40: 23% Women: <40: 74% |≥40: 26%	Overall retention rate: 73	YES
Guo (2020) [14]	China (urban)	Parallel RCT	PLWH aged ≥18; WeChat users; no difficulties in reading, listening and physical activities; currently not on any mental health services. Participant with depression scores: CES-D≥16	150 | 150	7.7	28.0 (5.8) | 28.6 (5.9)	88.7 | 84.7	YES
Jones (2016) [23]	Argentina (urban)	Full factorial pilot RCT	PLWH who were non-adherent to ART	60 | 60	49	All: 40.0 (8.6)	100 | 100	NO
Li (2017) [26]	China (rural)	Parallel cluster RCT	PLWH aged ≥18; many patients infected due to commercial plasma donations	257 | 265	55.2	50.0 (8.3) | 47.2 (9.1)	90.7 | 83.8	YES
Li (2021) [25]	China (urban)	Three-arm RCT	HIV-infected men who have sex with men; aged ≥18; QQ users; currently not on any mental health services. Exclusion: severe depression (PHQ≥20) or have suicidal thoughts	TGT-SN: 129 TGT-only: 139 Control: 136	0	TGT-SN: <30: 59.7%; >30: 40.3% TGT-only: <30: 65.5%; >30: 34.5% Control: <30: 59.6%; >30: 40.4%	TGT-SN: 86.8% TGT-only: 86.3% Control: 86.0%	NO
Meffert (2021) [32]	Kenya (urban)	Parallel RCT	PLWH aged ≥18; diagnosed with major depressive and posttraumatic stress disorders; being affected by gender-based violence	123 | 133	100	36.9 (9.4) | 37.0 (9.4)	72.4 | 88.0	YES
Nakimuli-Mpungu (2020) [15]	Uganda (rural)	Parallel cluster RCT	PLWH aged ≥18; currently on ART; no difficulties at listening or reading; not being at high risk of suicide; mild to moderate depression	578 | 562	53.7	38.9 (10.4) | 38.1 (11.5)	90.5 | 80.2	YES
Nyamathi (2012) [27]	India (rural)	Pilot parallel cluster RCT	Women with AIDS aged 18-45; CD4 count (≥100 cell/mm3 at baseline); currently on ART treatment (≥3 mo)	34 | 34	100	32.3 (5.3) | 30.1 (5.2)	100 | 100	NO
Peltzer (2012) [16]	South Africa (mixed)	Parallel RCT	PLWH aged ≥18 with ART adherence problem; currently on ART treatment	76 | 76	65.1	36.6 (9.4) | 37.1 (9.8)	96.1 | 97.4	NO
Petersen (2014) [28]	South Africa (peri-urban)	Pilot parallel RCT	PLWH aged ≥18; currently on ART; no difficulties at listening and speaking; no cognitive impairment. Being assessed as major depressive disorder by SCID-11	41 | 35	73.5	21-30: 23%; 31-40: 29%: 41-50: 29%; 51-59: 18% | 21-30: 47%; 31-40: 29%; 41-50: 12%; 51-59: 12%	41.5 | 48.6	NO
Sarna (2008) [29]	Kenya (urban)	Parallel RCT	PLWH aged ≥18; new to ART	116 | 118	63.7	37.3 (8.0) | 37 (7.8)	76.7 | 79.7	NO
Wang (2010) [30]	China (urban)	Parallel RCT	PLWH aged ≥18; current heroin users or have histories of heroin addiction; currently on ART (≥1 mo)	58 | 58	16.4	All: 36.7 (5.6)	86.2 | 82.8	NO
Williams (2014) [31]	China (urban)	Parallel RCT	PLWH who are currently on ART; ART adherence (<90%)	55 | 55	29.1	38 | 37 (SD not reported)	94.5 | 76.4	NO

ART – antiretroviral therapy, BDI – Beck's Depression Inventory, CES-D – Centre for Epidemiological Studies Depression, CG – control group, IG – intervention group, ITT – intention to treat analysis, NR – not reported, PHQ-9 – patient health questionnaire-9, PLWH – people living with HIV/AIDS, RCT – randomized control trial, Region – intervention conducted in rural or urban areas, SCID-11 – structural clinical interview for a DSM IV diagnosis, SD – standard deviation, TGT-SN – three good things (TGT) with electronic social networking, mo – months

*Studies used randomisation at the individual level, unless otherwise stated.

†Mean age and SD reported, unless otherwise stated; Intervention and control group information separated by the “|” symbol.

**Table 3:** Summary of included RCT findings of the intervention effects on depressive symptoms, grouped by intervention type

		Intervention group					Control group					
**First author (year)**	**Depression Tool (outcome assessment)**	**Number in group (total)**		Baseline depression, mean (SD)*		**Depression at Follow-up**†, mean (SD)*	**Number in group (total)**		Baseline depression, mean (SD)*		**Depression at follow-up**†, mean (SD)*	**Effect size [*P-*value]‡**
**Cognitive behavioural therapy**												
Chen (2018)[21]	CES-D, cut-off ≥16	21	44.3%		3 mo: 32.2%		20	42.0%		3 mo: 52.1%		OR 0.2, CI not reported [<0.01]
Guo (2020) [14]	CES-D	150	23.9 (6.4)		9 mo: 17.8 (10.6)		150	24.3 (6.9)		9 mo: 23.4 (11.2)		Between-group difference for mean change from baseline (95% CI) = -5.3 (-7.8, -2.8) [<0.001]
	PHQ-9	150	10.2 (4.5)		9 mo: 9.1 (5.1)		150	10.7 (5.1)		9 mo: 10.9 (5.4)		Mean difference between groups (95% CI) = -1.2 (-2.5, 0.1) [0.08]
Nakimuli-Mpungu (2020) [15]	MINI, major depression	578	0%		Post-treatment: 12 mo: 1%		562	0%		Post-treatment: 12 mo: 40%		Cluster-adjusted χ^2^ test = 6.32 [0.01]
Peltzer (2012) [16]	BDI-II	76	26.8 (22.2)		6 mo: 19.7 (19.3)		76	25.5(23.0)		6 mo: 19.2 (17.4)		F-value from ANOVA: 0.018 [0.894]
**Positive Psychology**												
Fawzi (2019)[22]	PHQ-9	M = 155 F = 199	NR		10 weeks: M = 0.8 (0.6) F = 0.8 (0.5)		M = 384 F = 519	NR		10 weeks: M = mean 0.9 (0.6) F = 0.9 (0.6)		Mean difference between groups (95% CI): M = -0.1 (-0.2, -0.03) [0.009] F = -0.1 (-0.2, -0.05) [0.0009]
Li (2021)[25]	CES-D, cut-off ≥16	TGT-SN = 129 TGT-only = 139	TGT-SN = 55.8% TGT-only = 51.1%		13 mo: TGT-SN = 45.5% TGT-only = 54.2%		136	61.0%		13 mo: 55.6%		AOR 11 mo: TGT-SN = 0.75(0.52 to 1.09) [0.131] TGT-only = 0.83(0.57 to 1.21) [0.332]
**Supportive**												
Jones (2016)[23]	BDI-II	60	4.4 (0.6)		9 mo: 3.3 (0.6)		60	4.2 (0.7)		9 mo: 1.4 (0.6)		t-value from generalized linear model: 2.26 [0.03]
Li (2017)[26]	Zung Self-Rating Depression Scale	257	22.3 (5.2)		NR		265	22.3 (5.5)		NR		Between-group difference for mean change from baseline (SE): -1.5 (0.5) [0.0006]
Nyamathi (2012)[27]	CES-D	34	NR		6 mo: 16.9 (9.6)		34	NR		6 mo: 10.6 (12.7)		β-value from linear regression model: 22.89 (SE 3.2); [0.001]
Sarna (2008)[29]	BDI-I	116	NR		18 mo: NR		118	NR		18 mo: NR		Median decrease in score (intervention vs control of 10 vs 6.5; [0.03]
Wang (2010)[30]	Self-rating Depression Scale	58	60.0 (13.8)		8 mo: 47.6 (14.8)		58	59.5 (12.9)		8 mo: 59.4 (15.0)		F-value from ANOVA: 5.58 [0.02]
Williams (2014)[31]	CES-D, cut-off ≥16	55	58%		12 mo: 40%		55	76%		12 mo:76%		NR [0.001]
**Interpersonal Therapy**												
Meffert (2021) [32]	MINI, major depression	123	100%		3 mo: 33.5%		133	100%		3 mo: 57.6%		OR 0.26 (0.11 to 0.60) [0.002]
Petersen (2014)[28]	PHQ-9	41	15.5 (4.5)		3 mo: 6.9 (4.1)		35	15.2(5.5)		3 mo: 11.1 (4.6)		F-value from ANOVA: 23.88 [<0.0001]

ANOVA – analysis of variance, AOR – adjusted odds ratio, BDI – Beck's Depression Inventory, CBT – cognitive behavioural therapy, CES-D – Centre for Epidemiological Studies Depression, MINI – mini international neuropsychiatric interview, NR – not reported, OR – odds ratio, PHQ-9 – patient health questionnaire-9, RCT – randomised controlled trial, SD – standard deviation, SE – standard error, mo – months

*Mean and SD reported, unless a diagnostic cut-off or tool was used, in which case the proportion is present.

†Follow-up from baseline, unless otherwise stated.

‡Results correspond to the follow-up period stated in the previous column, unless otherwise stated; effect size reported as proportion differences between groups.

### Intervention characteristics

The characteristics of interventions varied considerably, particularly in terms of the nature, delivery and intensity of the intervention (Table 2). Interventions were most commonly delivered by non-mental health nurses [21,29-31] or community workers (such as local health educators, lay HIV counsellors, women with education level above high school, or health workers infected with HIV and experienced in providing counselling services) [15,16,22,26-28,32]. In the remaining studies, deliverers included infectious disease physicians [23], peers via online platform or self-writing (a three arm RCT) [25] or an online platform delivering CBT courses [14]. All but three studies [16,29,30] provided information on the training and supervision of deliverers. Four trials [14-16,21] used CBT, with components including HIV education [15,16], identification of personal problems or concerns [15,16,21], and strategies or techniques to manage problems such as intrusive or depressive thoughts, stress, stigma or discrimination [14-16,21]. Two trials applied positive psychology [22,25], which focuses on positive aspects of life such as happiness and hope. One encouraged participants in the intervention group to identify values or good qualities in each other [22], the other encouraged participants to write down three good things to express gratitude and give daily feedback to others [25]. Two applied interpersonal therapy [28,32], which components including management of stigma, social isolation or intrusive thoughts [28], handling of interpersonal crisis [28,32] and build social skills [32]. The remaining 6 trials [23,26,27,29-31] were grouped as supportive interventions. One of these focused on strengthening connection within families and local communities to confront HIV-related challenges [26]. In the remaining five supportive interventions, common components included identification and discussion of challenges in antiretroviral therapy (ART) adherence and provision of techniques or personalized support to improve medication adherence.

**Table 2:** Characteristics of Interventions in included studies

First author (year)	Type of intervention; cultural adaptation	Level (L); Number of session (N); Duration interventions (D)	Mode (M); setting (S); provider of interventions (P); Outcome collectors (O)	Components in intervention and control groups	Supervisions or quality control of interventions
Chen (2018) [21]	CBT; Adapted based on previous qualitative interviews	L: Individual N: 3 sessions (60-90 min per session) D: 4 weeks	M: Face-to-face S: hospital P: Nurses O: NR	Relaxation techniques, cognitive/behaviour skills for mental health management, psychoeducation, & family support Control: usual HIV care	One intensive training week; feedback regarding the roleplay is given until nurses demonstrate sufficient fidelity; supervisors review nurses’ checklists of content and logs of progress after each session
Fawzi (2019) [22]	Positive psychology; adapted for use in Tanzania	L: Group N: 10 weekly sessions (30-35 h total) D: 10 weeks	M: face-to-face S: primary school P: HIV-infected community health workers O: NR	Identifying values/qualities in each other, develop knowledge related to sexual relationship & HIV, develop assertiveness and income-generating skills	2-week intensive training, supervisors review providers’ self-evaluation during training; research assistant examined the process of three sessions
Guo (2020) [14]	CBT; Materials translated into Chinese with formative research to enhance cultural relevance	L: Individual N: 12 sessions D: 3 mo	M: Online S: Online P: WeChat platform O: self-reported	Cognitive behavioural stress management and physical exercise in multimedia formats (images, audio, and essays) Control: usual care and nutrition brochures	Automated and instant information and feedback; phone calls made to identify difficulties of adherence and ensure correct use of the online platform
Jones (2016) [23]	Supportive; Materials translated and pilot tested in Argentina	L: Unknown N: Unknown D: Unknown	M: Face to face S: clinics P: infectious disease physicians O: NR	Providers using motivational interviews to help patients identify & overcome the challenge in ART adherence and break a bad habit Control: providers not active in interventions	Two workshops were organised to train providers using motivational interviews strategies and other skills such as using open-ended questions.
Li (2017) [26]	Multilevel supportive; Adapted to family-oriented traditions	L: Group (10-12 people), family & community N: 6 group sessions, 6 family activities, 3 community events & 10 reunion sessions D: 24 mo	M: face-to-face S: home and public places P: Local health educators O:NR	Group level (discussion of HIV challenges and coping techniques); family level (family integration); community (health fair, sporting activities, talent show) & 10 reunion sessions Control: standard HIV care plus weekly health education	Intensive trainings by research teams, multiple role-play simulation and evaluators are trained to assess fidelity according to evaluation checklist
Li (2021) [25]	Positive psychology; Unknown	L: Group (11-30 people per group) N: NA D: 1 mo	M: Online (TGT-SN); Self-writing (TGT-only) S: QQ online platforms P: self/peer support O: well-experienced filed workers	TGT-SN: write three good things, read, & give feedback on good things by others daily online TGT-only: write down three good things Control: weekly messages of mental health promotion information	Two MPH students monitor processes, deal with problems and remove negative information posted by participants; online meeting bi-weekly with the research author to ensure quality
Meffert (2021) [32]	Interpersonal therapy; Adapted via qualitative mental health needs assessment	L: Individual N: 12 sessions (one hour per session) D: 3 mo	M: face-to-face S: private rooms adjacent to clinics P: women completed high school	Personal symptoms review; interpersonal crisis management; social skills building	10-d training by experts in interpersonal therapies; weekly telephone supervisions during training and trials
Nakimuli-Mpungu (2020) [15]	CBT; Adapted via qualitative interviews with local community members	L: Group (10-12 people per group) N: 8 sessions D: 8 weeks	M: face-to-face S: primary health centres P: lay mental health workers O: NR	Education of HIV/AIDS & depression; personal problems sharing; skills for mental health problems management; techniques to deal with stigma or discrimination; financial skills to generate income Control: HIV and ART education	5-d intensive training by health workers who receive training from mental health specialists; supervision checklists used to guide training and assess competency; ongoing supervisions
Nyamathi (2012) [27]	Supportive; Unknown	L: individual N: 6 educational sessions plus weekly visit D: Unknown	M: face-to-face S: home P: Lay village women O: NR,	6 educational sessions: weekly visits by lay village women who provided support for ART adherence, healthy lifestyle adoption, and access to health services Control: 6 educational sessions plus monthly visited by lay village women who simply take records of ART adherence and medical outcomes. Both groups: monthly supply of 1 kg fruit.	Lay village women: 3-d training which includes didactic and mock sessions; ongoing supervision
Peltzer (2012) [16]	CBT; Unknown	L: Group (10 people per group) N: 3 sessions (one hour per session) D: 3 mo	M: face-to-face S: hospitals P: lay mental health workers and ART adherence counsellors O: trained interviewers	Medication education & identification of challenges to ART adherence; discussion about concerns & difficulties of medication adherence & solution sharing within groups. CG: Medical check-up monthly with physicians	Unknown
Petersen (2014) [28]	Interpersonal therapy; Adapted via qualitative interviews with PLWH in local areas	L: Group N: 8 sessions D: 8 weeks	M: face-to-face S: hospitals P: Lay HIV counsellors O: NR	Psychoeducation; techniques to manage stigma, social isolation, poverty, intrusive thoughts, and interpersonal conflicts. Control: Standard HIV care	4-d training delivered by psychologist and psychology trainees; ongoing weekly supervision at the first two months; then monthly supervision for the following months
Sarna (2008) [29]	Supportive; Unknown	L: Individual N: 48 visits D: 24 weeks	M: face-to-face S: clinics P: Nurses O: research assistants	Nurses observe & record ART dosage; identify challenges of ART adherence & give personalised support; support medicine provision Control: routine care	Unknown
Wang (2010) [30]	Supportive; Unknown	L: individual N: 4 home visits & bi-weekly telephone calls D: 8 mo	M: face-to-face and telephone S: home/via telephone P: Nurses O: NR	Education of HIV/AIDS and ART adherence; help to identify challenges & develop personalised plan for medication taking; provide some adherence techniques and mobilise family support Control: routine care	Unknown
Williams (2014) [31]	Supportive; Theory applied judged as appropriate and intervention was well known in China	L: individual N: 9 home visits D: 6 mo	M: face-to-face S: 47% at home and 53% in public places P: nurses and peer educators O: trained interviewers	Patient-directed approach in identification & discussion of challenges in medication adherence. Control: standard adherence support	Being trained to apply Freirian educational techniques during visits; notes of content and progress of visits made by nurses are reviewed to ensure fidelity

NR - not reported, mo – months

The control group similarly varied across studies (Table 2). Among the eleven parallel RCTs [14-16,21,26-32] and one three-arm RCT [25], seven included control groups where participants received usual HIV care or standardised adherence support provided in local countries or areas [16,21,28-32]. Three of these plus the remaining two studies included a control group where participants received additional care such as nutritional brochures [14], weekly message of psychoeducation [25], and HIV/AIDS health education or medication adherence in the format of courses [15,26,27].

The study which used a full factorial design [23] only presented depression outcomes for participants randomised to provider active and provider inactive interventions. Participants in provider inactive groups did not receive motivational interviews with health providers. Participants in the stepped-wedge trial [22] participants were randomly exposed to interventions in three chronological steps. Therefore, participants in control groups were those not receiving interventions in the first two steps.

### Effect of interventions on depression outcomes

Duration of study follow-up was generally short, ranging from two-and-a-half months [22] to 24 months [26] with just five studies following participants for at least one year (**Table 3**) [15,25,26,29,31]. Effect sizes were reported in diverse formats, including mean differences, between-group difference for mean change from baseline, standardised mean difference or odds ratio. In the 8 studies that included depressive symptoms as a primary outcome, of which four were restricted to people with depression at baseline [14,15,28,32], 7 found that the intervention was effective at reducing depressive symptoms more in participants in the intervention as compared to control arm [14,15,21,26-28,32]. However, the eighth study, which applied positive psychology via online groups or self-writing platforms, did not find an effect on depression symptoms [25].

Of the 6 studies [16,22,23,29-31] that included depression as a secondary outcome, five similarly reported a statistically significant positive effect of interventions, in terms of a greater reduction in depressive symptoms in participants in the intervention arm. The one study that ascertained presence of major depression at the trial end-point reported statistically significantly lower odds of depression in participants receiving the intervention vs control [31]. Only one study, a trial of face-to-face CBT in a hospital setting in people with ART adherence problems, found no statistically significant effect of the intervention on depressive symptoms after 6 months follow-up [16].

### Risk of bias within studies

**Figure 2:**
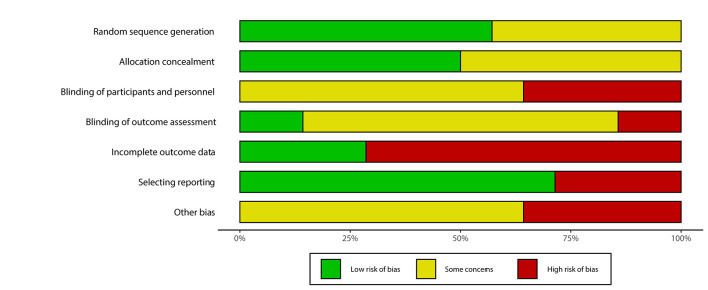
Summary bar plot of “risk of bias” assessment of included RCTs.

**Figure 3:**
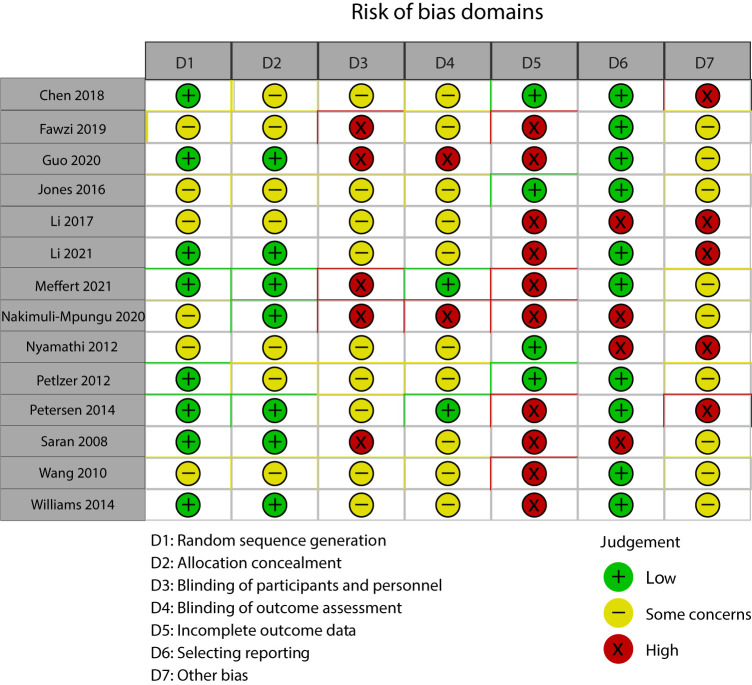
Traffic-light plot of “risk of bias” for each included study.

All but two studies [16,23] had at least one area of high risk of bias, and all studies had at least one area of concern due to incomplete reporting. As summarised in **Figures 2** and **3**, high risk of bias occurred in domains three to seven, with incomplete outcome data being the most common domain where high risk of bias was present. This was due to ten trials having high attrition rates (>10%) or differential attrition rates between groups [14,15,22,25,26,28-32]. Five trials were judged to have high risk of bias within the ‘other bias’ domain [21,25-28]. These included: lack of control for clustering [26,27]; no reporting of internal contamination of intervention (collected in just one trial [25], which suggested this may have led to an underestimate of effect); and small sample size [21,27,28] leading to insufficient power to detect statistically significant intervention effect. Direct quotes from studies or their protocols which help to support judgement of bias in each of the 7 domains are given in Appendix S3 in the **Online Supplementary Document**.

## DISCUSSION

The findings of our review suggest that non-mental health specialist delivered interventions may improve, or decrease the risk of developing, depression in PLWH in LMIC settings. In all but two study, effect sizes were statistically significant, with participants in the intervention arm likely to have lower or more improved depressive symptom scores (or lower odds of having major depression) than those in the control arm. However, there are a number of important caveats which limit the conclusions of this review. Existing studies are often small and markedly heterogeneous in terms of study population, intervention components, delivery, duration of follow-up and method of analysis, which affects comparability. Moreover, many studies had methodological limitations that may have increased the risk of bias and most studies assessed immediate or short-term outcomes only.

### Comparisons with existing literature

We found evidence of a statistically significant positive effect of interventions delivered by non-mental health specialists on depression in PLWH across four types of psychosocial intervention (CBT, positive psychology, interpersonal psychotherapy and supportive interventions). This effect was consistent despite considerable heterogeneity across study populations, intervention components, intervention intensity and type of non-mental health specialist deliverer. Whilst statistically significant effects on depression were observed in all but two of the 10 non-pilot RCTs, albeit with varying levels of statistical significance, it is unclear whether interventions had a clinically meaningful effect on depression, since this was not addressed in individual studies.

Our findings on the effects of CBT-based interventions align with a previous review, which found that CBT is effective in reducing common mental disorders in PLWH in LMIC settings irrespective of the deliverer being a trained mental health specialist [7]. It also suggests that in addition to being effective at reducing symptoms of common mental disorders in the general population in LMIC settings, delivery of CBT interventions by non-mental health specialists may also be an effective approach in PLWH [33]. Interventions focused on positive psychology and interpersonal psychotherapy and delivered by non-mental health specialists may also have an effect on depression. However, given the limited number of such studies, this is a gap that needs addressed in future studies. Our review also suggests that supportive interventions delivered by non-mental health specialists which have a key focus on ART adherence support may reduce depressive symptoms in PLWH. However, since depression was a secondary outcome in all but one of these studies [27], further RCTs with depression as a primary outcome are needed. Depression is associated with non-adherence to HIV medication, irrespective of country income level [34], and the findings from the studies included in the present review provide support for a bi-directional association, with improvement in ART adherence reducing the likelihood of depression, perhaps through improvement in quality of life [35]. Family and community integration and support formed the key component of the fifth supportive intervention [26], where depression was included as a primary outcome, with observed positive effects on depressive symptom scores. Involvement of family interaction or peer support has been highlighted in previous reviews as a potentially important component to include in trials of mental health in PLWH [8].

Although most of the included trials in our review evaluated interventions delivered face-to-face, the promising findings of the two studies delivered online [14,25] suggest that this approach may be worthy of further investigation. It does however rely on access to technology and internet, which may be a limitation in some settings.

### Strengths and limitations

Our review is the first to focus specifically on RCTs seeking to evaluate the effect of psychosocial interventions delivered by non-mental health specialists in resource-limited settings and is timely given the recent increase in the number of such studies. Our review benefits from robust methodology, including the use of a comprehensive electronic search strategy supplemented by some searching of the grey literature to help reduce publication bias. We used the PRISMA-Protocol guidance when developing the review methodology and two reviewers independently performed study screening, selection, data extraction and quality assessment.

Our review has some limitations, mostly due to the limitations of the identified studies themselves. The heterogeneity between studies, in terms of the study population, intervention type and components, intervention delivery and control group, reduces comparability of studies. Given this heterogeneity, it was not feasible, or appropriate, to conduct a meta-analysis and thus draw deductions about the effect size of such interventions as part of this review. Study populations differed markedly, with some including selected populations including women or men only, people with ART adherence problems, or current or prior heroin users. The selective nature of some study populations limits generalisability to other population sub-groups. The heterogeneity between included studies does limit the conclusions that can be drawn in this review. The diversity of interventions among a small number of studies precluded detailed comparisons of the effectiveness of different types of interventions on depression. However, this review was focused on determining whether interventions delivered by non-specialist mental health workers are effective in LMICs. It is interesting and promising that, despite the heterogeneity between studies, all but two studies did report a positive effect on the depression outcome. A key strength of this review is that it highlights important gaps in the literature to be addressed in future studies. The nature of the psychosocial interventions meant that it was difficult to implement and ensure that participants and outcome assessors were blind to intervention group. Lack of blinding could feasibly have introduced bias if knowing whether you received the intervention or not affected the measurement of depression. It is also unclear whether authors of the included studies sought to minimise contamination of comparison groups. However, treatment contamination would underestimate the effect sizes and thus underestimate the effect of interventions on depression outcomes [20]. In addition, only one study included information on the concurrent mental health services available to participants. The control groups were frequently described as receiving usual or routine care in the included studies. This again reduces comparability and limits generalisability of individual study findings given that routine care will likely differ between countries and between rural and urban areas. The studies identified in the present review were generally multi-component interventions which further complicates comparability across studies. Detailed descriptions of components and fidelity in the delivery would facilitate a deeper understanding of the intervention outcomes. Almost all studies assessed immediate or short-term outcomes and so there is little information on whether interventions are sustainable in the long-term. Moreover, given the lack of data on and discussion of the clinical meaningfulness of the observed effects on depression within the identified studies, this gap should be addressed in future trials to ensure studies clearly demonstrate not only whether an intervention effect is statistically significant, but also clinically meaningful. Finally, given the differences in analysis approaches across studies and in depression measurement, it was not possible to assess publication bias through construction of, and testing for asymmetry in, a funnel plot. It is possible that small negative RCTs may not have been published and hence included in our search. However, we did minimise the introduction of publication bias by review some grey literature and searching across a wide range of databases.

### Implications for research, policy and practice

Psychosocial interventions delivered by non-specialist mental health workers in LMICs may reduce depression in PLWH. The current evidence base does have important limitations, but the findings of our review suggest that this approach is promising and should continue to be investigated. As highlighted in the limitations section, there are a number of methodological improvements which future RCTs should aim to address. Future research should endeavour to avoid the common shortcomings of existing studies. In particular, future studies should ensure that loss to follow-up is minimal, outcome assessment is blinded to intervention allocation and contamination between intervention and control groups is reported. Future trials should also be adequately powered to detect statistically significant effects with a high level of precision. Supportive interventions and CBT were the two commonest type of interventions among the psychosocial interventions identified in our review, with interventions using positive psychology and interpersonal therapy approaches comparatively less studied, indicating a particular gap in the evaluation of the former. However, the relatively small number of studies identified in total indicates a need for further investigation of the effectiveness of each of these psychosocial intervention approaches, to determine their effectiveness in reducing depression in PLWH when delivered by non-mental health specialists. Additionally, RCTs should incorporate assessment of the fidelity of non-mental health specialist workers training and of intervention delivery. Moreover, given the relatively short follow-up period of existing RCTs, further research should establish the long-term sustainability of treatment and treatment effects. Whilst there is some evidence that, in the general population, non-specialist delivered psychological interventions are acceptable and feasible in LMICs [36], there is also evidence that the challenges faced by non-specialist mental health workers, including high workload and lack of funding support, as well as acceptability by participants, could negatively influence treatment sustainability [37]. Future qualitative studies would provide a deeper understanding of these potential real-world challenges. Due to a lack of economic evaluation of psychosocial interventions in PLWH in LIMICs [7,15] there is also a need to conduct cost-effectiveness analysis of mental health services delivered by mental health specialists vs lay health workers. Despite the further work needed in this area, the evidence from this review might inform guidelines of comprehensive HIV/AIDS management in resource-limited countries. Since depressive symptoms were related to ART adherence and development of AIDS, integration of mental health services delivered by lay health workers into current HIV care might help realize 95-95-95 targets (95% of PLWH diagnosed, 95% of PLWH diagnosed receive sustained ART, 95% of PLWH receiving sustained ART are virally suppressed) in 2030 in LMICs.

## CONCLUSION

To conclude, psychosocial interventions delivered by non-specialist mental health workers in PLWH in LMICs may be effective in reducing depressive symptoms or preventing depressive disorder in this vulnerable group. Existing RCTs do have some limitations that should be addressed in future studies. Qualitative studies and cost-effectiveness analysis of this intervention approach are also needed to gain deeper insight into the acceptability and value of such an approach and to inform further intervention development. In the meantime, public health bodies in LMIC settings should consider the potential benefit of non-specialist mental health delivery of interventions aimed at reducing depression in PLWH and the value of integrating such mental health services into current HIV care.

## Additional material


Online Supplementary Document
