# Supplementary material for: Psychosocial interventions for depression delivered by non-mental health specialists to people living with HIV/AIDS in low- and middle-income countries: A systematic review
Source: J Glob Health. 2022 Jun 11;12:04049. doi: 10.7189/jogh.12.04049 (PMC9185189; doi:10.7189/jogh.12.04049)
Supplement: Online Supplementary Document [file jogh-12-04049-s001.pdf]

Appendix 1 Details of electronic search strategies

Database: Ovid MEDLINE

1. exp HIV Infections/ (285670)

2. exp HIV/ (99735)

3. (hiv OR hiv1 OR hiv2 OR hiv infect* OR PLHIV OR HIV?AIDS OR AIDS).mp. (413944)

4. (“acquired immun?deficiency syndrome” OR “acquired immun?deficiency virus” OR “acquired immun?-deficiency syndrome” OR “acquired immun?-deficiency virus” OR “immun* deficiency virus” OR “immun* deficiency syndrome”).mp. (91611)

5. 1 OR 2 OR 3 OR 4 (428040)

6. exp Developing Countries/ (75433)

7. (Africa OR Asia OR Caribbean OR “West Indies” OR “South America” OR “Latin America” OR “Central America”).mp. (249152)

8. ((developing OR “less developed” OR “under developed” OR “underdeveloped” OR “middle income” OR “low* income”) adj (population* OR countr* OR nation* OR world OR econom$)).mp. (137932)

9. (low* adj (gdp OR gnp OR gross domestic OR gross national)).mp. (223)

10. (lmic OR lmics OR lamics OR lamic OR third world OR lami countr$).mp. (6383)

11. (transitional country OR transitional countries).mp. (144)

12. (Afghanistan OR Albania OR Algeria OR Angola OR Argentina OR Armenia OR Armenian OR "Agalega Islands" OR Azerbaijan OR Bangladesh OR Basutoland OR Benin OR Byelarus OR Byelorussian OR Belarus OR Belorussian OR Belorussia OR Belize OR Bhutan OR Bolivia OR Bosnia OR Botswana OR Brazil OR Bulgaria OR "Burkina Faso" OR "Burkina Fasso" OR Burundi OR Burma OR Cambodia OR Cameroon OR Cameroons OR Cameron OR Camerons OR "Cape Verde" OR "Cabo Verde" OR "Central African Republic" OR Chad OR China OR Colombia OR Comoros OR "Comoro Islands" OR Comoros OR "Costa Rica" OR "Cote d Ivoire" OR "Cote d'Ivoire" OR Croatia OR Cuba OR Congo OR Djibouti OR "Dominican Republic" OR Dominica OR "Equatorial Guinea" OR Eritrea OR Ethiopia OR "East Timor" OR "East Timur" OR Ecuador OR Eswatini OR Egypt OR "El Salvador" OR Fiji OR "French Somaliland" OR Gabon OR "Gabonese Republic" OR Gambia OR Gaza OR Georgia OR "Georgian Republic" OR Ghana OR "Gold Coast" OR Grenada OR Guatemala OR Guinea OR Grenadines OR Guinea-Bisseau OR Guiana OR Guyana OR Haiti OR Herzegovina OR Hercegovina OR Honduras OR Ifni OR "Ivory Coast" OR India OR Indonesia OR Iran OR Iraq OR Jamaica OR Jordan OR "Khmer Republic" OR Kampuchea OR Kazakhstan OR Kazakh OR Kenya OR Kiribati OR Korea OR Kosovo OR Kyrgyzstan OR "Kyrgyz Republic" OR Kirghizia OR Kyrgyz OR Kirghiz OR Kirgizstan OR Lao OR "Lao PDR" OR Laos OR Lebanon OR Lesotho OR Liberia OR Libya OR Mayotte OR Macedonia OR Madagascar OR Malagasy OR Malaysia OR Malaya OR Malay OR Malawi OR Mali OR "Mariana Islands" OR "Marshall Islands" OR Mauritania OR Mauritius OR Micronesia OR "Middle East" OR Maldives OR (Mexico not "New Mexico") OR Moldova OR Moldovia OR Moldovian OR Mongolia OR Montenegro OR Morocco OR Mozambique OR Myanmar OR Myanma OR Nyasaland OR Namibia OR "Navigator Island" OR "Navigator Islands" OR Nauru OR Nepal OR "Netherlands Antilles" OR "New Caledonia" OR "New Hebrides" OR Nicaragua OR Niger OR Nigeria OR Pakistan OR Palestine OR Panama OR "Papua New Guinea" OR Paraguay OR Peru OR Philippines OR Philipines OR Phillipines OR Phillippines OR Romania OR Rumania OR Roumania OR Russia OR Russian OR Rwanda OR Ruanda OR Rhodesia

OR "Saint Lucia" OR "St Lucia" OR "Saint Vincent" OR Sabah OR Sarawak OR "St Vincent" OR Samoa OR "Samoan Islands" OR "Sao Tome" OR Senegal OR Serbia OR Montenegro OR "Sierra Leone" OR "South Africa" OR "Sri Lanka" OR Ceylon OR "Solomon Islands" OR Somalia OR "Soviet Union" OR Somaliland OR "South Africa" OR Sudan OR "South Sudan" OR Suriname OR Surinam OR Swaziland OR Syria OR "Syrian Republic" OR Tajikistan OR Tadzhikistan OR Tadjikistan OR Tadzhik OR Tanzania OR Thailand OR "Timor Leste" OR Timor-Leste OR Togo OR Togolese OR Tonga OR Tunisia OR Turkey OR Turkmenistan OR Turkmen OR Tuvalu OR Uganda OR Ukraine OR USSR OR Uzbek OR "Union of Soviet Socialist Republics" OR "Upper Volta" OR Urundi OR Uzbekistan OR Venezuela OR Vanuatu OR Venezuela OR Vietnam OR "Viet Nam" OR "West Bank" OR Yemen OR Yugoslavia OR Zaire OR Zambia OR Zimbabwe).mp. (1491852)

13. 6 OR 7 OR 8 OR 9 OR 10 OR 11 OR 12 (1644428)

14. exp Depression/ (121458)

15. exp Depressive Disorder/ (110031)

16. (depress* disorder* OR Depress*).tw,kw. (408875)

17. exp Mental Health/ (39869)

18. (“mental health” OR “mental ill*” OR “mental disorder*” OR psychiatric OR “psychological health” OR wellbeing OR “well being” OR well-being).tw,kw. (376906)

19. exp Anxiety Disorders/ (79967)

20. exp Anxiety/ (86968)

21. (anxiety disorder$ OR anxi* OR overanxious OR fear).tw,kw. (224424)

22. exp Stress, Psychological/ (133339)

23. (stress* OR distress*).tw,kw. (812931)

24. 14 OR 15 OR 16 OR 17 OR 18 OR 19 OR 20 OR 21 OR 22 OR 23 (1644800)

25. exp Psychotherapy/ (197597)

26. exp Mental Health Services/ (96682)

27. exp Adaptation, Psychological/ (128264)

28. exp Cognitive Behavioral Therapy/ OR exp Mindfulness/ OR exp Meditation/ (31375)

29. exp Family Therapy/ (8875)

30. (psychotherap$ OR psychosocial OR psycho-social OR counsel* OR problem solving OR sociotherap*).mp. (314533)

31. ((Psycho-social OR “psycho social” OR psychosocial) adj3 (intervent$ OR therap$ OR treat$ OR manag$)).mp. (10411)

32. (Social adj1 (support OR system* OR skill*)).mp. (92157)

33. (socioenvironment$ adj1 (therap$ OR support OR intervent$)).mp. (464)

34. (support adj3 psycho).mp. (265)

35. exp Social Support/ (72014)

36. (‘self help” OR “peer support” OR “self care” OR “Support group*” OR “problem solving”).mp. (98485)

37. (Cbt OR Icbt OR “cognitive behavio?r therap*” OR “cognitive restructur$” OR “stress manage*” OR psychoed* OR relaxation OR mindfulness).mp. (122236)

38. (relaxation adj1 (techinique* OR therap$ OR training)).mp. (7214)

39. ((psycholog$ OR psychotherap$) adj3 (intervent$ OR therap$ OR treat$ OR manag$ OR adjustment* OR adaptation*)).mp. (118890)

40. ((education* OR behavio?r OR cogniti$) adj3 (intervent$ OR therap$ OR treat$ OR manag$)).mp. (114231)

41. (Interpersonal adj1 (therap$ OR treat$ OR psychotherap*)).mp. (1237)

42. (Coping adj1 (behavio* OR intervention* OR treat$ OR skill* OR strateg*)).mp. (16063)

43. ((Adaptive adj1 behavio*) OR family therap$ OR talking therap$).mp. (13992)

44. 25 OR 26 OR 27 OR 28 OR 29 OR 30 OR 31 OR 32 OR 33 OR 34 OR 35 OR 36 OR 37 OR 38 OR 39 OR 40 OR 41 OR 42 OR 43 (828928)

45. randomized controlled trial.pt. (516305)

46. controlled clinical trial.pt. (93895)

47. randomized.ti,ab. (459800)

48. drug therapy.fs. (2249292)

49. randomly.ab. (290454)

50. trial.ab. (448618)

51. groups.ab. (1794957)

52. 45 OR 46 OR 47 OR 48 OR 49 OR 50 OR 51 (4397799)

53. exp animals/ not humans.sh. (4753759)

54. 52 not 53 (3765451)

55. 5 and 13 and 24 and 44 and 54 (876)

Database: Cochrane

#1 MeSH descriptor: [HIV] explode all trees

#2 MeSH descriptor: [HIV Infections] explode all trees

#3 (hiv OR hiv1 OR hiv2 OR "hiv infect*" OR PLHIV OR HIV?AIDS OR AIDS):ti,ab,kw

#4 ("acquired immunodeficiency syndrome" OR "acquired immune deficiency syndrome" OR "acquired immune-deficiency syndrome" OR "acquired immuno deficiency syndrome" OR "human immunodeficiency virus" OR "human immune deficiency virus" OR "human immune-deficiency virus" OR "human immuno deficiency virus"):ti,ab,kw

#5 #1 OR #2 OR #3 #4

#6 MeSH descriptor: [Developing Countries] explode all trees

#7 (Africa OR Asia OR Caribbean OR "West Indies" OR "South America" OR "Latin America" OR "Central America"):ti,ab,kw

#8 ((developing OR "less developed" OR "under developed" OR underdeveloped OR "middle income" OR "low* income") NEXT (population* OR countr* OR nation* OR world OR econom*)):ti,ab,kw

#9 ("low* NEXT (gdp OR gnp OR "gross domestic" OR "gross national")"):ti,ab,kw

#10 (lmic OR lmics OR lamics OR lamic OR "third world" OR "lami countr*"):ti,ab,kw

#11 "transitional countries":ti,ab,kw

#12 (Afghanistan OR Albania OR Algeria OR Angola OR Argentina OR Armenia OR Armenian OR "Agalega Islands" OR Azerbaijan OR Bangladesh OR Basutoland OR Benin OR Byelarus OR Byelorussian OR Belarus OR Belorussian OR Belorussia OR Belize OR Bhutan OR Bolivia OR Bosnia OR Botswana OR Brazil OR Bulgaria OR "Burkina Faso" OR "Burkina Fasso" OR Burundi OR Burma OR Cambodia OR Cameroon OR Cameroons OR Cameron OR Camerons OR "Cape Verde" OR "Cabo Verde" OR "Central African Republic" OR Chad OR China OR Colombia OR Comoros OR "Comoro Islands" OR Comoros OR "Costa Rica" OR "Cote d Ivoire" OR "Cote d'Ivoire" OR Croatia OR Cuba OR Congo OR Djibouti OR "Dominican Republic" OR Dominica OR "Equatorial Guinea" OR Eritrea OR Ethiopia OR "East Timor" OR "East Timur" OR Ecuador OR Eswatini OR Egypt OR "El Salvador" OR Fiji OR "French Somaliland" OR Gabon OR "Gabonese Republic" OR Gambia OR Gaza OR Georgia OR "Georgian Republic" OR Ghana OR "Gold Coast" OR Grenada OR Guatemala OR Guinea OR Grenadines OR Guinea-Bisseau OR Guiana OR Guyana OR Haiti OR Herzegovina OR Hercegovina OR Honduras OR Ifni OR "Ivory Coast" OR India OR Indonesia OR Iran OR Iraq OR Jamaica OR Jordan OR "Khmer Republic" OR Kampuchea OR Kazakhstan OR Kazakh OR Kenya OR Kiribati OR Korea OR Kosovo OR Kyrgyzstan OR "Kyrgyz Republic" OR Kirghizia OR Kyrgyz OR Kirghiz OR Kirgizstan OR Lao OR "Lao PDR" OR Laos OR Lebanon OR Lesotho OR Liberia OR Libya OR Mayotte OR Macedonia OR Madagascar OR Malagasy OR Malaysia OR Malaya OR Malay OR Malawi OR Mali OR "Mariana Islands" OR "Marshall Islands" OR Mauritania OR Mauritius OR Micronesia OR "Middle East" OR Maldives OR (Mexico NOT "New Mexico") OR Moldova OR Moldovia OR Moldovian OR Mongolia OR Montenegro OR Morocco OR Mozambique OR Myanmar OR Myanma OR Nyasaland OR Namibia OR "Navigator Island" OR "Navigator Islands" OR Nauru OR Nepal OR "Netherlands Antilles" OR "New Caledonia" OR "New Hebrides" OR Nicaragua OR Niger OR Nigeria OR Pakistan OR Palestine OR Panama OR "Papua New Guinea" OR Paraguay OR Peru OR Philippines OR Philipines OR Phillipines OR Phillippines OR Romania OR Rumania OR Roumania OR Russia OR Russian OR Rwanda OR Ruanda OR Rhodesia OR "Saint Lucia" OR "St Lucia" OR "Saint Vincent" OR Sabah OR Sarawak OR "St Vincent" OR Samoa OR "Samoan Islands" OR "Sao Tome" OR Senegal OR Serbia OR Montenegro OR "Sierra Leone" OR "South Africa" OR "Sri Lanka" OR Ceylon OR "Solomon Islands" OR Somalia OR "Soviet Union" OR Somaliland OR "South Africa" OR Sudan OR "South Sudan" OR Suriname OR Surinam OR Swaziland OR Syria OR "Syrian Republic" OR Tajikistan OR Tadzhikistan OR Tadjikistan OR Tadzhik OR Tanzania OR Thailand OR "Timor Leste" OR Timor-Leste OR Togo OR Togolese OR Tonga OR Tunisia OR Turkey OR Turkmenistan OR Turkmen OR Tuvalu OR Uganda OR Ukraine OR USSR OR Uzbek OR "Union of Soviet Socialist Republics" OR "Upper Volta" OR Urundi OR Uzbekistan OR Venezuela OR Vanuatu OR Venezuela OR Vietnam OR "Viet Nam" OR "West Bank" OR Yemen OR Yugoslavia OR Zaire OR Zambia OR Zimbabwe

):ti,ab,kw

#13 #6 OR #7 OR #8 OR #9 OR #10 OR #11 OR #12

#14 MeSH descriptor: [Depression] explode all trees

#15 MeSH descriptor: [Depressive Disorder] explode all trees

#16 ("depress* disorder*" OR Depress*):ti,ab,kw

#17 MeSH descriptor: [Mental Health] explode all trees

#18 MeSH descriptor: [Anxiety] explode all trees

#19 MeSH descriptor: [Anxiety Disorders] explode all trees

#20 ("anxiety disorder*" OR anxi* OR overanxious OR fear):ti,ab,kw

#21 MeSH descriptor: [Stress, Psychological] explode all trees

#22 (stress* OR distress*):ti,ab,kw

#23 ("mental health" OR "mental ill*" OR "mental disorder*" OR psychiatric OR "psychological health" OR "well being"):ti,ab,kw

#24 #14 OR #15 OR #16 OR #17 OR #18 OR #19 OR #20 OR #21 OR #22 OR #23

#25 MeSH descriptor: [Psychotherapy] explode all trees

#26 MeSH descriptor: [Mental Health Services] explode all trees

#27 MeSH descriptor: [Adaptation, Psychological] explode all trees

#28 MeSH descriptor: [Behavior Therapy] explode all trees

#29 (psychotherap* OR psychosocial OR psycho-social OR counsel* OR "problem solving" OR sociotherap*):ti,ab,kw

#30 (("Psycho social" OR psychosocial OR "psycho-social") NEAR/3 (intervent* OR therap* OR treat* OR manag*)):ti,ab,kw

#31 (Social NEXT (support OR system* OR skill*)):ti,ab,kw

#32 (socioenvironment* NEXT therap*):ti,ab,kw

#33 (support NEAR/3 psycho):ti,ab,kw

#34 MeSH descriptor: [Social Support] explode all trees

#35 ("self help" OR "peer support" OR "self care" OR "Support group*" OR "problem solving"):ti,ab,kw

#36 (Cbt OR Icbt OR "cognitive behavio?r therap*" OR "cognitive restructur*" OR "stress manage*" OR psychoed* OR relaxation OR mindfulness):ti,ab,kw

#37 (relaxation NEAR/1 (techinique* OR therap* OR training)):ti,ab,kw

#38 ((psycholog* OR psychotherap*) NEAR/3 (intervent* OR therap* OR treat* OR manag* OR adjustment* OR adaptation*)):ti,ab,kw

#39 ((education* OR behavio?r OR cogniti*) NEAR/3 (intervent* OR therap* OR treat* OR manag*)):ti,ab,kw

#40 (Interpersonal NEXT (therap* OR treat* OR psychotherap*)):ti,ab,kw

#41 (Coping NEAR/1 (behavio* OR intervention* OR treat* OR skill* OR strateg*)):ti,ab,kw

#42 ((Adaptive NEAR/1 behavio*) OR "family therap*" OR "talking therap*"):ti,ab,kw

#43 #25 OR #26 OR #27 OR #28 OR #29 OR #30 OR #31 OR #32 OR #33 OR #34 OR #35 OR #36 OR #37 OR #38 OR #39 OR #40 OR #41 OR #42

#44 #5 and #13 and #24 and #43 (714)

Database: Embase

1. exp Human immunodeficiency virus infection/ (382307)

2. exp Human immunodeficiency virus/ (198952)

3. exp acquired immune deficiency syndrome/ (141473)

4. (hiv OR hiv1 OR hiv2 OR hiv infect* OR PLHIV OR HIV?AIDS OR AIDS).mp. (509824)

5. (acquired immun?deficiency syndrome OR acquired immun?deficiency virus OR acquired immun?-deficiency syndrome OR acquired immun?-deficiency virus OR immun* deficiency syndrome OR human immun* deficiency virus).mp. (146580)

6. 1 OR 2 OR 3 OR 4 OR 5 (604763)

7. exp developing country/ (94954)

8. (Africa OR Asia OR Caribbean OR West Indies OR South America OR Latin America OR Central America).mp. (364169)

9. ((developing OR less developed OR under developed OR underdeveloped OR middle income OR low* income) adj (population* OR countr* OR nation* OR world OR econom$)).mp. (183588)

10. (low* adj (gdp OR gnp OR gross domestic OR gross national)).mp. (374)

11. (lmic OR lmics OR lamics OR lamic OR third world OR lami countr$).mp. (10591)

12. (transitional country OR transitional countries).mp. (238)

13. (Afghanistan OR Albania OR Algeria OR Angola OR Argentina OR Armenia OR Armenian OR Azerbaijan OR Bangladesh OR Benin OR Byelarus OR Byelorussian OR Belarus OR Belorussian OR Belorussia OR Belize OR Bhutan OR Bolivia OR Bosnia OR Herzegovina OR Hercegovina OR Botswana OR Brazil OR Bulgaria OR Burkina Faso OR Burkina Fasso OR Upper Volta OR Burundi OR Urundi OR Cambodia OR Khmer Republic OR Kampuchea OR Cameroon OR Cameroons OR Cameron OR Camerons OR Cape Verde OR Cabo Verde OR Central African Republic OR Chad OR China OR Colombia OR Comoros OR Comoro Islands OR Comoros OR Mayotte OR Congo OR Zaire OR Costa Rica OR Cote d Ivoire OR Cote d'Ivoire OR Ivory Coast OR Croatia OR Cuba OR Djibouti OR French Somaliland OR Dominica OR Dominican Republic OR East Timor OR East Timur OR Timor Leste OR Timor-Leste OR Ecuador OR Egypt OR El Salvador OR Equatorial Guinea OR Eritrea OR Ethiopia OR Fiji OR Gabon OR Gabonese Republic OR Gambia OR Gaza OR Georgia OR Georgian Republic OR Ghana OR Gold Coast OR Grenada OR Guatemala OR Guinea OR Guinea-Bisseau OR Guiana OR Guyana OR Haiti OR Honduras OR India OR Indonesia OR Iran OR Iraq OR Jamaica OR Jordan OR Kazakhstan OR Kazakh OR Kenya OR Kiribati OR Korea OR Kosovo OR Kyrgyzstan OR Kyrgyz Republic OR Kirghizia OR Kyrgyz OR Kirghiz OR

Kirgizstan OR Lao OR Lao PDR OR Laos OR Lebanon OR Lesotho OR Basutoland OR Liberia OR Libya OR Macedonia OR Madagascar OR Malagasy OR Malaysia OR Malaya OR Malay OR Sabah OR Sarawak OR Malawi OR Nyasaland OR Mali OR Marshall Islands OR Mauritania OR Mauritius OR Agalega Islands OR (Mexico not New Mexico) OR Micronesia OR Middle East OR Maldives OR Moldova OR Moldovia OR Moldovian OR Mongolia OR Montenegro OR Morocco OR Ifni OR Mozambique OR Myanmar OR Myanma OR Burma OR Namibia OR Nauru OR Nepal OR Netherlands Antilles OR New Caledonia OR Nicaragua OR Niger OR Nigeria OR Mariana Islands OR Pakistan OR Palestine OR Panama OR Papua New Guinea OR Paraguay OR Peru OR Philippines OR Philipines OR Phillipines OR Phillippines OR Romania OR Rumania OR Roumania OR Russia OR Russian OR Rwanda OR Ruanda OR Saint Lucia OR St Lucia OR Saint Vincent OR St Vincent OR Grenadines OR Samoa OR Samoan Islands OR Navigator Island OR Navigator Islands OR Sao Tome OR Senegal OR Serbia OR Montenegro OR Sierra Leone OR South Africa OR Sri Lanka OR Ceylon OR Solomon Islands OR Somalia OR Somaliland OR South Africa OR Sudan OR South Sudan OR Suriname OR Surinam OR Swaziland OR Eswatini OR Syria OR Syrian Republic OR Tajikistan OR Tadzhikistan OR Tadjikistan OR Tadzhik OR Tanzania OR Thailand OR Togo OR Togolese OR Tonga OR Tunisia OR Turkey OR Turkmenistan OR Turkmen OR Tuvalu OR Venezuela OR Uganda OR Ukraine OR USSR OR Soviet Union OR Union of Soviet Socialist Republics OR Uzbekistan OR Uzbek OR Vanuatu OR New Hebrides OR Venezuela OR Vietnam OR Viet Nam OR West Bank OR Yemen OR Yugoslavia OR Zambia OR Zimbabwe OR Rhodesia).mp. (2047800)

14. 7 OR 8 OR 9 OR 10 OR 11 OR 12 OR 13 (2293172)

15. exp depression/ (488090)

16. (depress* disorder* OR Depress*).tw,kw. (639860)

17. exp mental health/ (164733)

18. (mental health OR mental ill* OR mental disorder* OR psychiatric OR psychological health OR wellbeing OR well being OR well-being).tw,kw. (584341)

19. exp anxiety disorder/ (250387)

20. exp anxiety/ (216929)

21. (anxiety disorder$ OR anxi* OR overanxious OR fear).tw,kw. (382232)

22. exp mental stress/ (84296)

23. (stress* OR distress*).tw,kw. (1279816)

24. 15 OR 16 OR 17 OR 18 OR 19 OR 20 OR 21 OR 22 OR 23 (2615022)

25. exp psychodynamic psychotherapy/ OR exp interpersonal psychotherapy/ OR exp psychotherapy/ (255709)

26. exp mental health service/ (56715)

27. exp coping behavior/ OR exp Meditation/ OR exp mindfulness/ (81491)

28. exp behavior therapy/ OR exp cognitive behavioral therapy/ OR exp cognitive therapy/ (91820)

29. exp family therapy/ (13213)

30. exp social support/ (94292)

31. (psychotherap$ OR psychosocial OR psycho-social OR counsel* OR problem solving OR sociotherap*).mp. (516885)

32. ((Psychosocial OR psycho social OR psycho-social) adj3 (intervent$ OR therap$ OR treat$ OR manag$)).mp. (18323)

33. (Social adj1 (support OR system* OR skill*)).mp. (116919)

34. (support adj3 psycho).mp. (723)

35. (self help OR peer support OR self care OR Support group* OR problem solving).mp. (149737)

36. (Cbt OR Icbt OR cognitive behavio?r therap* OR cognitive restructur$ OR stress manage* OR psychoed* OR relaxation OR mindfulness).mp. (204433)

37. (relaxation adj1 (techinique* OR therap$ OR training)).mp. (12281)

38. ((psycholog$ OR psychotherap$) adj3 (intervent$ OR therap$ OR treat$ OR manag$ OR adjustment* OR adaptation*)).mp. (50518)

39. ((education* OR behavio?r OR cogniti$) adj3 (intervent$ OR therap$ OR treat$ OR manag$)).mp. (190663)

40. (Interpersonal adj1 (therap$ OR treat$ OR psychotherap*)).mp. (2113)

41. (Coping adj1 (behavio* OR intervention* OR treat$ OR skill* OR strateg*)).mp. (69403)

42. ((Adaptive adj1 behavio*) OR family therap$ OR talking therap$).mp. (69760)

43. 25 OR 26 OR 27 OR 28 OR 29 OR 30 OR 31 OR 32 OR 33 OR 34 OR 35 OR 36 OR 37 OR 38 OR 39 OR 40 OR 41 OR 42 (1182660)

44.. clinical trial/ (994628)

45 randomized controlled trial/ (633143)

46. randomization/ (89120)

47. single blind procedure/ (41037)

48. double blind procedure/ (178963)

49. crossover procedure/ (65349)

50. placebo/ (359531)

51. prospective study/ (643448)

52. randomi?ed controlled trial$.tw. (243751)

53. rct.tw. (39499)

54. (random$ adj2 allocat$).tw. (45040)

55. ((single OR double OR treble OR triple) adj blind$).tw. (240651)

56. placebo$.tw. (317754)

57. 44 OR 45 OR 46 OR 47 OR 48 OR 49 OR 50 OR 51 OR 52 OR 53 OR 54 OR 55 OR 56 (2299030)

58. 6 and 14 and 24 and 43 and 57 (399)

Database: Global Health

1. exp HIV infections/ (97096)

2. exp human immunodeficiency viruses/ (107140)

3. exp acquired immune deficiency syndrome/ (22894)

4. (hiv OR hiv1 OR hiv2 OR hiv infect* OR PLHIV OR HIV?AIDS OR AIDS).mp. (120074)

5. (acquired immun?deficiency syndrome OR acquired immun?deficiency virus OR acquired immun?-deficiency syndrome OR acquired immun?-deficiency virus OR immun* deficiency syndrome OR human immun* deficiency virus).mp. (23902)

6. 1 OR 2 OR 3 OR 4 OR 5 (120884)

7. exp Developing Countries/ (693473)

8. (Africa OR Asia OR Caribbean OR West Indies OR South America OR Latin America OR Central America).mp. (767709)

9. ((developing OR less developed OR under developed OR underdeveloped OR middle income OR low* income) adj (population* OR countr* OR nation* OR world OR econom$)).mp. (704638)

10. (low* adj (gdp OR gnp OR gross domestic OR gross national)).mp. (73)

11. (lmic OR lmics OR lamics OR lamic OR third world OR lami countr$).mp. (17581)

12. (transitional country OR transitional countries).mp. (89)

13. (Afghanistan OR Albania OR Algeria OR Angola OR Argentina OR Armenia OR Armenian OR Azerbaijan OR Bangladesh OR Benin OR Byelarus OR Byelorussian OR Belarus OR Belorussian OR Belorussia OR Belize OR Bhutan OR Bolivia OR Bosnia OR Herzegovina OR Hercegovina OR Botswana OR Brazil OR Bulgaria OR Burkina Faso OR Burkina Fasso OR Upper Volta OR Burundi OR Urundi OR Cambodia OR Khmer Republic OR Kampuchea OR Cameroon OR Cameroons OR Cameron OR Camerons OR Cape Verde OR Cabo Verde OR Central African Republic OR Chad OR China OR Colombia OR Comoros OR Comoro Islands OR Comoros OR Mayotte OR Congo OR Zaire OR Costa Rica OR Cote d Ivoire OR Cote d'Ivoire OR Ivory Coast OR Croatia OR Cuba OR Djibouti OR French Somaliland OR Dominica OR Dominican Republic OR East Timor OR East Timur OR Timor Leste OR Timor-Leste OR Ecuador OR Egypt OR El Salvador OR Equatorial Guinea OR Eritrea OR Ethiopia OR Fiji OR Gabon OR Gabonese Republic OR Gambia OR Gaza OR Georgia OR Georgian Republic OR Ghana OR Gold Coast OR Grenada OR Guatemala OR Guinea OR Guinea-Bisseau OR Guiana OR Guyana OR Haiti OR Honduras OR India OR Indonesia OR Iran OR Iraq OR Jamaica OR Jordan OR Kazakhstan OR Kazakh OR Kenya OR Kiribati OR Korea OR Kosovo OR Kyrgyzstan OR Kyrgyz Republic OR Kirghizia OR Kyrgyz OR Kirghiz OR Kirgizstan OR Lao OR Lao PDR OR Laos OR Lebanon OR Lesotho OR Basutoland OR Liberia OR Libya OR Macedonia OR Madagascar OR Malagasy OR Malaysia OR Malaya OR Malay OR Sabah OR Sarawak OR Malawi OR Nyasaland OR Mali OR Marshall Islands OR Mauritania OR Mauritius OR Agalega Islands OR (Mexico not New Mexico) OR Micronesia OR Middle East OR Maldives OR Moldova OR Moldovia OR Moldovian OR Mongolia OR Montenegro OR Morocco OR Ifni OR Mozambique OR Myanmar OR Myanma OR Burma OR Namibia OR Nauru OR Nepal OR Netherlands Antilles OR New Caledonia OR Nicaragua OR Niger OR Nigeria OR Mariana Islands OR Pakistan OR Palestine OR Panama OR Papua New Guinea OR Paraguay OR Peru OR Philippines OR Philipines OR Phillipines OR Phillippines OR Romania OR Rumania OR Roumania OR Russia OR Russian OR Rwanda OR Ruanda OR Saint Lucia OR St Lucia OR Saint Vincent OR St Vincent OR Grenadines OR Samoa OR Samoan Islands OR Navigator Island OR Navigator Islands OR Sao Tome OR Senegal OR Serbia OR Montenegro OR Sierra Leone OR South Africa OR Sri Lanka OR Ceylon OR Solomon Islands OR Somalia OR Somaliland OR South Africa OR Sudan OR South Sudan OR Suriname OR Surinam OR Swaziland OR Eswatini OR Syria OR Syrian Republic OR Tajikistan OR Tadzhikistan OR Tadjikistan OR Tadzhik OR Tanzania OR Thailand OR

Togo OR Togolese OR Tonga OR Tunisia OR Turkey OR Turkmenistan OR Turkmen OR Tuvalu OR Venezuela OR Uganda OR Ukraine OR USSR OR Soviet Union OR Union of Soviet Socialist Republics OR Uzbekistan OR Uzbek OR Vanuatu OR New Hebrides OR Venezuela OR Vietnam OR Viet Nam OR West Bank OR Yemen OR Yugoslavia OR Zambia OR Zimbabwe OR Rhodesia).mp. (753298)

14. 7 OR 8 OR 9 OR 10 OR 11 OR 12 OR 13 (849513)

15. exp depression/ (22350)

16. (depress* disorder* OR Depress*).mp. (40683)

17. exp mental health/ (21719)

18. (mental health OR mental ill* OR mental disorder* OR psychiatric OR psychological health OR wellbeing OR well being OR well-being).mp. (73813)

19. 15 OR 16 OR 17 OR 18 (93791)

20. exp psychotherapy/ (2902)

21. family counselling.sh. (42)

22. (psychotherap$ OR psychosocial OR psycho-social OR counsel* OR problem solving OR sociotherap*).mp. (39323)

23. ((Psychosocial OR psycho social OR psycho-social) adj3 (intervent$ OR therap$ OR treat$ OR manag$)).mp. (934)

24. (Social adj1 (support OR system* OR skill*)).mp. (8875)

25. (support adj3 psycho).mp. (51)

26. (self help OR peer support OR self care OR Support group* OR problem solving).mp. (6607)

27. (Cbt OR Icbt OR cognitive behavio?r therap* OR cognitive restructur$ OR stress manage* OR psychoed* OR relaxation OR mindfulness).mp. (6630)

28. (relaxation adj1 (techinique* OR therap$ OR training)).mp. (692)

29. ((psycholog$ OR psychotherap$) adj3 (intervent$ OR therap$ OR treat$ OR manag$ OR adjustment* OR adaptation*)).mp. (3037)

30. ((education* OR behavio?r OR cogniti$) adj3 (intervent$ OR therap$ OR treat$ OR manag$)).mp. (15410)

31. (Interpersonal adj1 (therap$ OR treat$ OR psychotherap*)).mp. (86)

32. (Coping adj1 (behavio* OR intervention* OR treat$ OR skill* OR strateg*)).mp. (2875)

33. ((Adaptive adj1 behavio*) OR family therap$ OR talking therap$).mp. (354)

34. mental health service.mp. (591)

35. (Meditation OR mindfulness OR psychodynamic psychotherap* OR family therap*).mp. (995)

36. 20 OR 21 OR 22 OR 23 OR 24 OR 25 OR 26 OR 27 OR 28 OR 29 OR 30 OR 31 OR 32 OR 33 OR 34 OR 35 (72802)

37. randomized controlled trial.mp. OR exp randomized controlled trials/ (42780)

38. clinical trial.mp. OR exp clinical trials/ (62853)

39. randomization.mp. (3809)

40. ((double OR single) adj1 blind procedure).mp. (10)

41. prospective study.mp. (16671)

42. randomi?ed controlled trial$.tw. (48331)

43. rct.tw. (3241)

44. (random$ adj2 allocat$).tw. (4767)

45. ((single OR double OR treble OR triple) adj blind$).tw. (19882)

46. 37 OR 38 OR 39 OR 40 OR 41 OR 42 OR 43 OR 44 OR 45 (100554)

47. 6 and 14 and 19 and 36 and 46 (88)

Database: Global index medicus

tw:(tw:((tw:("depress* disorder*" OR depress* OR "mental health" OR "mental ill*" OR "mental disorder*" OR psychiatric OR "psychological health" OR wellbeing OR "well being" OR well-being OR "anxiety disorder*" OR anxi* OR overanxious OR fear OR stress* OR distress*)) AND (tw:(hiv OR hiv1 OR hiv2 OR "hiv infect*" OR plhiv OR hiv OR aids OR "acquired immunodeficiency syndrome" OR "acquired immune deficiency syndrome" OR "acquired immune-deficiency syndrome" OR "acquired immuno deficiency syndrome" OR "human immunodeficiency virus" OR "human immune deficiency virus" OR "human immune-deficiency virus" OR "human immuno deficiency virus"))) AND (type_of_study:("clinical_trials"))) （76）

Database: APA PsycInfo

1. exp AIDS/ OR exp HIV/ (43409)

2. (hiv OR hiv1 OR hiv2 OR hiv infect* OR PLHIV OR HIV?AIDS OR AIDS).mp. (74957)

3. (acquired immun?deficiency syndrome OR acquired immun?deficiency virus OR acquired immun?-deficiency syndrome OR acquired immun?-deficiency virus OR immun* deficiency virus OR immun* deficiency syndrome).mp. (7658)

4. 1 OR 2 OR 3 (75238)

5. exp Developing Countries/ (5598)

6. (Africa OR Asia OR Caribbean OR West Indies OR South America OR Latin America OR Central America).mp. (40779)

7. ((developing OR less developed OR under developed OR underdeveloped OR middle income OR low* income) adj (population* OR countr* OR nation* OR world OR econom$)).mp. (18240)

8. (low* adj (gdp OR gnp OR gross domestic OR gross national)).mp. (46)

9. (lmic OR lmics OR lamics OR lamic OR third world OR lami countr$).mp. (2114)

10. (transitional country OR transitional countries).mp. (66)

11. (Afghanistan OR Albania OR Algeria OR Angola OR Argentina OR Armenia OR Armenian OR Azerbaijan OR Bangladesh OR Benin OR Byelarus OR Byelorussian OR Belarus OR Belorussian OR Belorussia OR Belize OR Bhutan OR Bolivia OR Bosnia OR Herzegovina OR Hercegovina OR Botswana OR Brazil OR Bulgaria OR Burkina Faso OR Burkina Fasso OR Upper Volta OR Burundi OR Urundi OR Cambodia OR Khmer Republic OR Kampuchea OR Cameroon OR Cameroons OR Cameron OR Camerons OR Cape Verde OR Cabo Verde OR Central African Republic OR Chad OR China OR Colombia OR Comoros OR Comoro Islands OR Comoros OR Mayotte OR Congo OR Zaire OR Costa Rica OR Cote d Ivoire OR Cote d'Ivoire OR Ivory Coast OR Croatia OR Cuba OR Djibouti OR French Somaliland OR Dominica OR Dominican Republic OR East Timor OR East Timur OR Timor Leste OR Timor-Leste OR Ecuador OR Egypt OR El Salvador OR Equatorial Guinea OR Eritrea OR Ethiopia OR Fiji OR Gabon OR Gabonese Republic OR Gambia OR Gaza OR Georgia OR Georgian Republic OR Ghana OR Gold Coast OR Grenada OR Guatemala OR Guinea OR Guinea-Bisseau OR Guiana OR Guyana OR Haiti OR Honduras OR India OR Indonesia OR Iran OR Iraq OR Jamaica OR Jordan OR Kazakhstan OR Kazakh OR Kenya OR Kiribati OR Korea OR Kosovo OR Kyrgyzstan OR Kyrgyz Republic OR Kirghizia OR Kyrgyz OR Kirghiz OR Kirgizstan OR Lao OR Lao PDR OR Laos OR Lebanon OR Lesotho OR Basutoland OR Liberia OR Libya OR Macedonia OR Madagascar OR Malagasy OR Malaysia OR Malaya OR Malay OR Sabah OR Sarawak OR Malawi OR Nyasaland OR Mali OR Marshall Islands OR Mauritania OR Mauritius OR Agalega Islands OR (Mexico not New Mexico) OR Micronesia OR Middle East OR Maldives OR Moldova OR Moldovia OR Moldovian OR Mongolia OR Montenegro OR Morocco OR Ifni OR Mozambique OR Myanmar OR Myanma OR Burma OR Namibia OR Nauru OR Nepal OR Netherlands Antilles OR New Caledonia OR Nicaragua OR Niger OR Nigeria OR Mariana Islands OR Pakistan OR Palestine OR Panama OR Papua New Guinea OR Paraguay OR Peru OR Philippines OR Philipines OR Phillipines OR Phillippines OR Romania OR Rumania OR Roumania OR Russia OR Russian OR Rwanda OR Ruanda OR Saint Lucia OR St Lucia OR Saint Vincent OR St Vincent OR Grenadines OR Samoa OR Samoan Islands OR Navigator Island OR Navigator Islands OR Sao Tome OR Senegal OR Serbia OR Montenegro OR Sierra Leone OR South Africa OR Sri Lanka OR Ceylon OR Solomon Islands OR Somalia OR Somaliland OR South Africa OR Sudan OR South Sudan OR Suriname OR Surinam OR Swaziland OR Eswatini OR Syria OR Syrian Republic OR Tajikistan OR Tadzhikistan OR Tadjikistan OR Tadzhik OR Tanzania OR Thailand OR Togo OR Togolese OR Tonga OR Tunisia OR Turkey OR Turkmenistan OR Turkmen OR Tuvalu OR Venezuela OR Uganda OR Ukraine OR USSR OR Soviet Union OR Union of Soviet Socialist Republics OR Uzbekistan OR Uzbek OR Vanuatu OR New Hebrides OR Venezuela OR Vietnam OR Viet Nam OR West Bank OR Yemen OR Yugoslavia OR Zambia OR Zimbabwe OR Rhodesia).mp. (238007)

12. 5 OR 6 OR 7 OR 8 OR 9 OR 10 OR 11 (262260)

13. exp "Depression (Emotion)"/ OR exp Major Depression/ (158487)

14. (depress* disorder* OR Depress*).mp. (375097)

15. exp Mental Health/ (68437)

16. (mental health OR mental ill* OR mental disorder* OR psychiatric OR psychological health OR wellbeing OR well being OR well-being).mp. (579728)

17. exp Anxiety Disorders/ (53081)

18. exp Anxiety/ (74746)

19. (anxiety disorder$ OR anxi* OR overanxious OR fear).mp. (308432)

20. exp Distress/ OR exp Psychological Stress/ (30987)

21. (stress* OR distress*).mp. (364872)

22. 13 OR 14 OR 15 OR 16 OR 17 OR 18 OR 19 OR 20 OR 21 (1190825)

23. exp Psychotherapy/ (204675)

24. exp Mental Health Services/ (42288)

25. exp adjustment/ OR exp mindfulness-based interventions/ (53130)

26. exp Cognitive Therapy/ OR exp Cognitive Behavior Therapy/ (34842)

27. exp Family Therapy/ (22071)

28. exp Social Support/ (36949)

29. (psychotherap$ OR psychosocial OR psycho-social OR counsel* OR problem solving OR sociotherap*).mp. (471720)

30. ((Psychosocial OR psycho social OR psycho-social) adj3 (intervent$ OR therap$ OR treat$ OR manag$)).mp. (13169)

31. (Social adj1 (support OR system* OR skill*)).mp. (98106)

32. (socioenvironment$ adj1 therap$).mp. (12)

33. (support adj3 psycho).mp. (206)

34. (self help OR peer support OR self care OR Support group* OR problem solving).mp. (101809)

35. (Cbt OR Icbt OR cognitive behavio?r therap* OR cognitive restructur$ OR stress manage* OR psychoed* OR relaxation OR mindfulness).mp. (74042)

36. (relaxation adj1 (techinique* OR therap$ OR training)).mp. (5129)

37. ((psycholog$ OR psychotherap$) adj3 (intervent$ OR therap$ OR treat$ OR manag$ OR adjustment* OR adaptation*)).mp. (70826)

38. ((education* OR behavio?r OR cogniti$) adj3 (intervent$ OR therap$ OR treat$ OR manag$)).mp. (121439)

39. (Interpersonal adj1 (therap$ OR treat$ OR psychotherap*)).mp. (3030)

40. (Coping adj1 (behavio* OR intervention* OR treat$ OR skill* OR strateg*)).mp. (59611)

41. ((Adaptive adj1 behavio*) OR family therap$ OR talking therap$).mp. (41366)

42. 23 OR 24 OR 25 OR 26 OR 27 OR 28 OR 29 OR 30 OR 31 OR 32 OR 33 OR 34 OR 35 OR 36 OR 37 OR 38 OR 39 OR 40 OR 41 (873871)

43. Treatment Effectiveness Evaluation/ (25051)

44. exp Treatment Outcomes/ (124449)

45. Placebo/ (5786)

46. Followup Studies/ (12380)

47. (placebo* OR random* OR comparative stud*).tw. (243378)

48. (clinical adj3 trial*).tw. (37396)

49. (research adj3 design*).tw. (34511)

50. (evaluat* adj3 stud*).tw. (66058)

51. (prospectiv* adj3 stud*).tw. (34187)

52. ((singl* OR doubl* OR trebl* OR tripl*) adj3 (blind* OR mask*)).tw. (26775)

53. 43 OR 44 OR 45 OR 46 OR 47 OR 48 OR 49 OR 50 OR 51 OR 52 (502179)

54. 4 and 12 and 22 and 42 and 53 (385)

Database: Web of knowledge

#14: #13 AND #12 AND #8 AND #7 AND #1 (888)

#13: TS= clinical trial* OR TS=research design OR TS=comparative stud* OR TS=evaluation stud* OR TS=controlled trial* OR TS=follow-up stud* OR TS=prospective stud* OR TS=random* OR TS=placebo* OR TS=(single blind*) OR TS=(double blind*) (5,201,623)

#12: #11 OR #10 OR #9 (1,052,634)

#11: TS= (socioenvironment* NEAR/3 therap*) OR TS= (support NEAR/3 psycho*) OR TS= (relaxation NEAR/1 (techniqu* OR therap* OR training)) OR TS= ((psycholog* OR psychotherap*) NEAR/3 (intervent* OR therap* OR treat* OR manag* OR adjustment* OR adaptation*)) OR TS= ((education* OR behavio?r OR cogniti*) NEAR/3 (intervent* OR therap* OR treat* OR manag*)) OR TS= (Interpersonal NEAR/0 (therap* OR treat* OR psychotherap*)) OR TS= (Coping NEAR/0 (behavio* OR intervention* OR treat* OR skill* OR strateg*)) OR TS=((Adaptive NEAR/0 behavio*) OR "family therap*" OR "talking therap*") (252,136)

#10: TS= (("Psycho social" OR psychosocial OR "psycho-social") NEAR/3 (intervent* OR therap* OR treat* OR manag*)) (17,080)

#9: TS= (psychotherap* OR psychosocial OR psycho-social OR counsel* OR "problem solving" OR sociotherap* OR "self help" OR "peer support" OR "self care" OR "Support group*" OR "problem solving" OR Cbt OR Icbt OR "cognitive behavio?r therap*" OR "cognitive restructur*" OR "stress manage*" OR psychoed* OR relaxation OR mindfulness) (877,385)

#8: TS= ("depress* disorder*" OR Depress* OR "mental health" OR "mental ill*" OR "mental disorder*" OR psychiatric OR "psychological health" OR wellbeing OR "well being" OR well-being OR "anxiety disorder*" OR anxi* OR overanxious OR fear OR stress* OR distress*) (3,331,762)

#7: #6 OR #5 OR #4 OR #3 OR #2 (16,877,485)

#6: ALL= ("transitional countries" OR lmic OR lmics OR lamics OR lamic OR "third world" OR "lami countr*") (25,700)

#5: CU=(Afghanistan OR Albania OR Algeria OR Angola OR Argentina OR Armenia OR Armenian OR "Agalega Islands" OR Azerbaijan OR Bangladesh OR Basutoland OR Benin OR Byelarus OR Byelorussian OR Belarus OR Belorussian OR Belorussia OR Belize OR Bhutan OR Bolivia OR Bosnia OR Botswana OR Brazil OR Bulgaria OR "Burkina Faso" OR "Burkina Fasso" OR Burundi OR Burma OR Cambodia OR Cameroon OR Cameroons OR Cameron OR Camerons OR "Cape Verde" OR "Cabo Verde" OR "Central African Republic" OR Chad OR China OR Colombia OR Comoros OR "Comoro Islands" OR Comoros OR "Costa Rica" OR "Cote d Ivoire" OR "Cote d'Ivoire" OR Croatia OR Cuba OR Congo OR Djibouti OR "Dominican Republic" OR Dominica OR "Equatorial Guinea" OR Eritrea OR Ethiopia OR "East Timor" OR "East Timur" OR Ecuador OR Eswatini OR Egypt OR "El Salvador" OR Fiji OR "French Somaliland" OR Gabon OR "Gabonese Republic" OR Gambia OR Gaza OR Georgia OR "Georgian Republic" OR Ghana OR "Gold Coast" OR Grenada OR Guatemala OR Guinea OR Grenadines OR

Guinea-Bisseau OR Guiana OR Guyana OR Haiti OR Herzegovina OR Hercegovina OR Honduras OR Ifni OR "Ivory Coast" OR India OR Indonesia OR Iran OR Iraq OR Jamaica OR Jordan OR "Khmer Republic" OR Kampuchea OR Kazakhstan OR Kazakh OR Kenya OR Kiribati OR Korea OR Kosovo OR Kyrgyzstan OR "Kyrgyz Republic" OR Kirghizia OR Kyrgyz OR Kirghiz OR Kirgizstan OR Lao OR "Lao PDR" OR Laos OR Lebanon OR Lesotho OR Liberia OR Libya OR Mayotte OR Macedonia OR Madagascar OR Malagasy OR Malaysia OR Malaya OR Malay OR Malawi OR Mali OR "Mariana Islands" OR "Marshall Islands" OR Mauritania OR Mauritius OR Micronesia OR "Middle East" OR Maldives OR (Mexico NOT "New Mexico") OR Moldova OR Moldovia OR Moldovian OR Mongolia OR Montenegro OR Morocco OR Mozambique OR Myanmar OR Myanma OR Nyasaland OR Namibia OR "Navigator Island" OR "Navigator Islands" OR Nauru OR Nepal OR "Netherlands Antilles" OR "New Caledonia" OR "New Hebrides" OR Nicaragua OR Niger OR Nigeria OR Pakistan OR Palestine OR Panama OR "Papua New Guinea" OR Paraguay OR Peru OR Philippines OR Philipines OR Phillipines OR Phillippines OR Romania OR Rumania OR Roumania OR Russia OR Russian OR Rwanda OR Ruanda OR Rhodesia OR "Saint Lucia" OR "St Lucia" OR "Saint Vincent" OR Sabah OR Sarawak OR "St Vincent" OR Samoa OR "Samoan Islands" OR "Sao Tome" OR Senegal OR Serbia OR Montenegro OR "Sierra Leone" OR "South Africa" OR "Sri Lanka" OR Ceylon OR "Solomon Islands" OR Somalia OR "Soviet Union" OR Somaliland OR "South Africa" OR Sudan OR "South Sudan" OR Suriname OR Surinam OR Swaziland OR Syria OR "Syrian Republic" OR Tajikistan OR Tadzhikistan OR Tadjikistan OR Tadzhik OR Tanzania OR Thailand OR "Timor Leste" OR Timor-Leste OR Togo OR Togolese OR Tonga OR Tunisia OR Turkey OR Turkmenistan OR Turkmen OR Tuvalu OR Uganda OR Ukraine OR USSR OR Uzbek OR "Union of Soviet Socialist Republics" OR "Upper Volta" OR Urundi OR Uzbekistan OR Venezuela OR Vanuatu OR Venezuela OR Vietnam OR "Viet Nam" OR "West Bank" OR Yemen OR Yugoslavia OR Zaire OR Zambia OR Zimbabwe

) (16,749,464)

#4: TS= (low* NEAR/0 (gdp OR gnp OR "gross domestic" OR "gross national")) (480)

#3: TS=((developing OR "less developed" OR "under developed" OR underdeveloped OR "middle income" OR "low* income") NEAR/0 (population* OR countr* OR nation* OR world OR econom*)) OR ALL=(lmic OR lmics OR lamics OR lamic OR "third world" OR "lami countr*" OR "transitional countries") (250,335)

#2: CU=(Africa OR Asia OR Caribbean OR "West Indies" OR "South America" OR "Latin America" OR "Central America") (391,768)

#1: TS=(hiv OR hiv1 OR hiv2 OR "hiv infect*" OR PLHIV OR HIV OR AIDS OR "acquired immunodeficiency syndrome" OR "acquired immune deficiency syndrome" OR "acquired immune-deficiency syndrome" OR "acquired immuno deficiency syndrome" OR "human immunodeficiency virus" OR "human immune deficiency virus" OR "human immune-deficiency virus" OR "human immuno deficiency virus") (919,736)

Database: Clinical Trials

40 Studies found for: hiv OR hiv1 OR hiv2 OR PLHIV OR HIV OR AIDS OR "acquired immunodeficiency syndrome" OR "acquired immune deficiency syndrome" OR "acquired immune-deficiency syndrome" | depression OR anxiety OR stress | Psychotherapy OR "Mental Health Services" OR "psychoeducation" OR "Behavior Therapy" OR "Social support" OR "Peer support" OR "Relaxation" OR "Counselling" OR "Coping" OR "stress management" | Adult, Older Adult

Appendix 2: Reasons for excluding studies

**Author (Year)**	**Reasons for exclusion**
Abas et al (2018)	Deliveries: it involves psychologists and psychiatrists
Achterbergh el al (2017)	Country: based in Amsterdam, not a LMIC
Actrn (2012)	Country: based in Australia, not a LMIC
Adejumo et al (2016)	Study design: not an RCT, a case-control study
Adina et al (2017)	Deliveries: CBT treatment was delivered by a doctoral candidate in counselling psychology
Akena, Kuteesa and Alinaitwe (2016)	Study design: not an RCT, Editorial on Safren's study
Andersen et al (2018)	Deliveries: delivered by clinical psychologists, psychiatrists, and a psychiatric nurse.
Anonymous (2018)	Outcomes: depression was not assessed and based in USA
Anonymous (2018)	Study design: not an RCT, description of a programme
Antoni et al (1991)	Deliveries: intervention conducted by psychologists
Antoni et al (2000)	Country: based in the USA, not a LMIC
Antoni et al (2000)	Country: based in the USA, not a LMIC
Antoni et al (2000)	Deliveries: intervention delivered by persons having advanced training in clinical psychology
Antoni et al (2002)	Outcomes: depression was not assessed and based in USA
Antoni et al (2006)	Deliveries: intervention delivered by persons having advanced training in clinical psychology
Antoni et al (2008)	Deliveries: intervention delivered by persons having advanced training in clinical psychology
Attonito et al (2019)	Country: based in the USA, not a LMIC
Augustinavicius et al (2020)	Study design: not an RCT, secondary analysis of RCT data in a caregiver-child dyad study
Berg et al (2009)	Country: based in the USA, not a LMIC
Berger et al (2008)	Country: based in Switzerland, not a LIMC

Blashill et al (2017)	Country: based in the USA, not a LMIC
Bogart er al (2018)	Country: based in the USA, not a LMIC
Bormann and Carrico (2009)	Country: based in the USA, not a LMIC
Brandt et al (2019)	Country: based in the USA, not a LMIC
Brazier, Mulkins and Verhoef (2006)	Country: based in Canada, not a LMIC
Bremner et al (2016)	Country: based in the USA, not a LMIC
Brown et al (2011)	Country: based in the USA, not a LMIC
Brown et al (2019)	Country: based in the USA, not a LMIC
Carey et al (2019)	Deliveries: conducted by Psychologist and based in USA
Carneiro et al (2019)	Outcomes: depression was not assessed
Carrico et al (2005)	Deliveries: CBT delivered by a licensed psychologist or certified psychiatrist
Carrico et al (2005)	Deliveries: conducted by Psychologist and based in USA
Carrico et al (2006)	Deliveries: CBT delivered by a licensed psychologist or certified psychiatrist
Carrico et al (2015)	Outcomes: depression was not assessed and based in USA
Chattopadhyay et al (2017)	Deliveries: unclear deliveries
Chaudhury et al (2016)	Population: depression outcomes were only measured in children
Chesney et al (2018)	Country: based in the USA, not a LMIC
Chhatre et al (2013)	Country: based in the USA, not a LMIC
Chibanda et al (2011)	Population: Not PLWHIV
Chibanda et al (2016)	Population: Not PLWHIV
Côté and Pepler (2002)	Outcomes: depression was not assessed
Creswell et al (2009)	Outcomes: depression was not assessed and based in USA
Cruess et al (1999)	Country: based in the USA, not a LMIC
Cruess et al (2000)	Country: based in the USA, not a LMIC
Cruess et al （2000）	Deliveries: Intervention delivered by psychology graduates and conducted in the USA
Dale et al (2016)	Deliveries: conducted by Psychologist and based in USA
Dianatinasab et al (2018)	Intervention: physical exercise
Doerfler and Goodfellow (2006)	Outcomes: depression was not assessed

Duncan et al (2021)	Country: based in the USA, not a LMIC
Ekstrand et al (2020)	Outcomes: depression was not assessed
Eller (1995)	Country: based in the USA, not a LMIC
Eller et al (2013)	Country: Included 10 USA sites, South Africa and South America, results were not displayed separately for LMICs
Fan et al (2020)	Study design: a study protocol of an RCT
Field and Kruger (2008)	Deliveries: unclear deliveries
Futterman et al (2010)	Population: pregnant women
Gayner et al (2012)	Country: based in Canada, not a LMIC
Graham et al (2020)	Outcomes: depression was not assessed
Green et al (2010)	Interventions: deliver palliative care service and not an RCT
Grelotti et al (2017)	Country: based in the USA, not a LMIC
Han et al (2020)	Deliveries: delivered by workers who were trained to acquire the level 2 certificate
Han et al (2020)	Deliveries: a dataset of a RCCT, however it is delivered by workers who were trained to acquire the level 2 certificate.
Hansen et al (2015)	Deliveries: intervention delivered by two clinical psychologists and two clinical social workers. Also, set in the USA.
Hartson et al (2016)	Outcomes: depression was not assessed
Hassanpourdehkordi et al (2019)	Outcomes: depression was not assessed
Hayes-Larson et al (2017)	Study design: a cohort study not an RCT
Heckman et al (2006)	Country: based in the USA, not a LMIC
Heckman et al (2013)	Country: based in the USA, not a LMIC
Heckman et al (2017)	Country: based in the USA, not a LMIC
Heinemann et al (2017)	Population: pregnant women
Hersch et al (2013)	Outcomes: depression was not assessed and based in USA
Hightow-Weidman et al (2017)	Country: based in the USA, not a LMIC
Himelhoch et al (2013)	Country: based in the USA, not a LMIC
Hosseinian, Tabatabaei and Ravaei (2011)	Outcomes: depression was not assessed
Husbands et al (1994)	Country: based in Canada, not a LMIC

Inouye, Flannelly and Flannelly (2001)	Country: based in the USA, not a LMIC
Ironson et al (2013)	Country: based in the USA, not a LMIC
Isrctn (2012)	Country: based in Norway, not a LMIC
Jalali et al (2019)	Deliveries: intervention delivered by certified schema therapists
Jam et al (2010)	Outcomes: depression was not assessed
Jensen et al （2013）	Deliveries: intervention delivered by psychologist
Johnson et al (2011)	Country: based in the USA, not a LMIC
Johnson et al (2015)	Country: based in the USA, not a LMIC
Jones et al (2014)	Country: based in the USA, not a LMIC
Jones et al (2018)	Population: pregnant women
Jones et al (2019)	Outcomes: Depression was only measured at pre-intervention but not post intervention
Kaaya et al (2013)	Population: pregnant women
Kalichman (2005)	Country: based in the USA, not a LMIC
Katz et al (1996)	Population: HIV/AIDS education, participants were not infected by HIV
Kelly et al (1993)	Country: based in the USA, not a LMIC
Knettel et al (2019)	Study design: a qualitative study
Koblin et al (2012)	Country: based in the USA, not a LMIC
Koopman et al (2002)	Outcomes: depression was not assessed and based in USA
Kraaij et al (2010)	Country: based in Netherlands, not a LMIC
Kuloor, Kumari and Metri (2018)	Interventions: physical exercises
Lechner et al (2003)	Country: based in the USA, not a LMIC
Lee, Jong and Gay (2020)	Country: based in the USA, not a LMIC
Li et al (2010)	Deliveries: unclear deliveries
Li et al (2011)	Outcomes: it did not display the depression outcomes separately for HIV patients and their families.
Li et al (2011)	Deliveries: unclear deliveries
Li et al (2012)	Deliveries: delivered by trained intervention facilitators
Li et al (2019)	Study design: not an RCT, a secondary analysis of an RCT
Liu et al (2018)	Outcomes: depression was not assessed

Lovejoy (2012)	Deliveries: Clinical psychologist also based in USA
Lovejoy and Heckman (2014)	Deliveries: Clinical psychologist also based in USA
Lovejoy and Heckman (2015)	Deliveries: Clinical psychologist also based in USA
Lovejoy et al (2012)	Country: based in the USA, not a LMIC
Lowthe et al (2016)	Study design: not an RCT, a qualitative study
Lowther et al (2015)	Outcomes: depression was not assessed
Lowther et al (2018)	Outcomes: depression was not assessed
Lowther et al (2018)	Outcomes: depression was not assessed
Lutgendorf et al (1997)	Deliveries: intervention delivered by psychologist also USA based
Lutgendorf et al (1998)	Deliveries: intervention delivered by psychologist, also in USA
Madhombiro et al (2019)	Outcomes: depression was not assessed
Mann (2001)	Outcomes: depression was not assessed
Mao et al (2019)	Outcomes: depression was not assessed
Markowitz et al (1995)	Country: based in USA, not LMICs
Markowitz et al (1998)	Country: set in the USA, not a LIMC. Also, intervention by trained therapists.
Markowitz et al (2000)	Deliveries: intervention delivered by trained therapists
Masquillier et al (2014)	Outcomes: general mental health (depression and anxiety) outcomes were available; however, depression was not assessed specifically
McCain et al (2003)	Country: based in the USA, not a LMIC
McIntyre et al (2018)	Study design: not an RCT, a single-arm trial
Meade et al (2010)	Outcomes: depression was not assessed
Miles et al (2003)	Country: based in the USA, not a LMIC
Mills (2014)	Deliveries: intervention delivered by mental health workers with diploma level and degree training in psychiatric
Mirzaeidoostan, Zargar and Payam (2019)	Outcomes: depression was not assessed
Mitrani et al （2010）	Outcomes: depression was not assessed
Mitrani et al (2012)	Country: based in the USA, not a LMIC
Moghadam et al (2018)	Outcomes: depression was not assessed
Moghanloo et al (2019)	Language: Persion language - Google translate did not assess depression scores

Molassiotis et al (2002)	Outcomes: depression was not assessed
Moosa and Jeenah (2012)	Outcomes: depression was not assessed
Moskowitz et al (2016)	Country: based in the USA, not a LMIC
Moskowitz et al (2017)	Country: based in the USA, not a LMIC
Mulder et al (1994)	Country: based in Netherlands, not a LMIC
Murphy et al (2002)	Deliveries: intervention led by a cognitive-behavioural psychologist and a psychiatric nurse
Nakimuli-Mpungu et al (2014)	Deliveries: Intervention given by mental health workers
Nakimuli-Mpungu et al (2015)	Deliveries: intervention delivered by mental health workers with a mental health diploma or degree
Nakimuli-Mpungu et al (2017)	Study design: a study protocol of an RCT
Nakimuli-Mpungu et al (2019)	Outcome: depression was measured only at baseline
Nobakht et al (2018)	Deliveries: CBT delivered by an MS counselling midwifery student with a training in CBT
Nwobi et al (2018)	Deliveries: conducted by therapist
Nyamathi et al (2001)	Country: based in the USA, not a LMIC
Nyamathi et al (2012)	Outcomes: depression outcomes were not available
Nyamathi et al (2013)	Outcomes: depression outcomes were not available
Nyamayaro et al (2020)	Study design: not an RCT, case Series
O'Cleirigh et al (2018)	Deliveries: intervention by psychologists also based in USA
Olley (2006)	Deliveries: counselling given by trained counsellors
Pacella et al (2016)	Country: based in the USA, not a LMIC
Pence et al (2015)	Study design: not an RCT, a descript study
Pokhrel et al (2018)	Study design: not an RCT, participants in the intervention group were purposively selected
Poudel et al (2015)	Outcomes: depression was not assessed
Pyne et al (2011)	Country: based in the USA, not a LMIC
Ramirez-Garcia et al (2020)	Country: based in Canada, not a LMIC
Ransom et al (2008)	Country: set in the USA. Also, intervention delivered by trained and experienced therapists.
Rao et al (2009)	Deliveries: Intervention delivered by specialist (licensed art therapist) also set in USA

Remien et al (2010)	Study design: an abstract from a Conference
Robinson, Mathews and Witek-Janusek (2003)	Country: based in the USA, not a LMIC
Rotheram-Borus et al (2014)	Population: pregnant women
Sacks et al (2011)	Country: based in the USA, not a LMIC
Safren et al (2009)	Country: based in the USA, not a LMIC
Safren et al (2012)	Country: based in the USA, not a LMIC
Safrenet al (2015)	Country: USA, not LMICs,
Salmoirago-Blotcher et al (2017)	Country: based in the USA, not a LMIC
Scharer et al (2019)	Outcomes: depression was not assessed and based in USA
Schnall et al (2018)	Country: based in the USA, not a LMIC
SeyedAlinaghi et al (2012)	Outcomes: SCL-90 psychological score added up so not able to differentiate those that had depression from other mental illness
Sikkema et al (2004)	Country: USA based and intervention delivered by mental health specialists
Sikkema et al (2005)	Country: USA based and intervention delivered by mental health specialists
Sikkema et al (2006)	Outcomes: depression was not assessed and based in USA
Sikkema et al (2018)	Outcomes: depression was not assessed
Silveira et al (2014)	Outcomes: depression was not assessed
Simoni et al (2013)	Country: based in the USA, not a LMIC
Simoni et al (2015)	Outcomes: depression was not assessed
Skeie et al (2012)	Outcomes: depression was not assessed
Smith et al (2020)	Population: Not PLWHIV
Stein et al (2007)	Country: based in the USA, not a LMIC
Stockton et al (2020)	Study design: not an RCT, a single-arm trial
Surilena et al (2014)	Outcome: general mental health outcome not specific for depression
Swendeman et al (2015)	Study design: not an RCT, a single-arm trial
Tobin et al (2017)	Country: based in the USA, not a LMIC
Tshabalala and Visser (2011)	Deliveries: intervention delivered by trained people
Uebelacker et al (2016)	Deliveries: intervention given by psychologists

Verdeli et al (2012)	Study design: a book chapter, there is no primary data
Vian et al (2018)	Outcomes: depression was assessed only at baseline
Vidrine et al (2006)	Country: based in the USA, not a LMIC
Vidrine et al (2015)	Country: based in the USA, not a LMIC
Wagner et al (2010)	Outcomes: depression was not assessed
Wagner et al （2017）	Intervention: integrating antidepressant treatment into HIV care
Wang et al (2015)	Outcomes: depression was not assessed
Weaver et al (2009)	Country: based in the USA, not a LMIC
Webb et al (2018)	Country: based in the USA, not a LMIC
Weber et al (2004)	Deliveries: Intervention given by psychotherapist, also based in Switzerland
Weiss et al (2003)	Deliveries: intervention delivered by trained therapists.
Weston et al (2012)	Study design: a meeting/ conference abstract. Not enough information for data extraction and study appraisal
Wu et al (2017)	Country: based in the USA, not a LMIC
Yang et al (2018)	Study design: not an RCT, a single-arm trial
Ye et al (2018)	Outcomes: depression was not assessed
Zanetti et al (2016)	Intervention: physical exercise
Zhu et al (2019)	Study design: not an RCT, secondary analysis of RCT data
Zisook et al (1998)	Intervention: antidepressant
Znoj et al (2010)	Country: set in Switzerland, not a LMIC

Appendix S3

Chen (2018)

Bias	Judgement	Support for judgement
**Random sequence generation**	Low risk	Quote: “participants were randomized to either the control or intervention arm using a computer-randomized number in recruitment sequence”
**Allocation concealment**	Some concerns	No information provided.
**Blinding of participants and personnel**	Some concerns	Method of blinding is not mentioned.
**Blinding of outcome assessment**	Some concerns	Method of blinding is not mentioned.
**Incomplete outcome data**	Low risk	Comment: (0/22) no one was lost to follow up in intervention group, 2/20(10%) in control group did not complete follow up. Relatively low attrition rate.
**Selecting reporting**	Low risk	No evidence of selective reporting. Expected outcomes were reported in this article.
**Other bias**	High risk	Small sample size and results might not be powerful.

Fawzi (2019)

Bias	Judgement	Support for judgement
**Random sequence generation**	Some concerns	Quote: "people living with HIV (hereafter referred to as Change Agents) were randomly allocated to receive the NAMWEZA intervention in one of three chronological steps". Comment: The term "randomized" is mentioned, no further information to support the judgement.
**Allocation concealment**	Some concerns	No information provided.
**Blinding of participants and personnel**	High risk	Quote:" it was not possible to 'blind' group facilitators or study participants when a specific group was receiving the intervention".
**Blinding of outcome assessment**	Some concerns	No information provided.
**Incomplete outcome data**	High risk	Quote: "overall follow-up rate was 73%. Limited retention of study participants over time may potentially bias our findings. Comment: high attrition rate.
**Selecting reporting**	Low risk	Expected outcomes were reported in this article.
**Other bias**	Some concerns	Concurrent treatment participants might receive and contamination of intervention were not documented.

Guo (2020)

Bias	Judgement	Support for judgement
**Random sequence generation**	Low risk	Quote: "Allocation to the treatment group was carried out by a computer-generated randomization list with a block size of 4, using SAS software version 9.4 (SAS Institute, Inc)."
**Allocation concealment**	Low risk	Quote: "Allocation to the treatment group was carried out by a computer-generated randomization list with a block size of 4, using SAS software version 9.4 (SAS Institute, Inc)."
**Blinding of participants and personnel**	High risk	Quote: "By the nature of the trial design, neither the research staff nor the participants were blinded to the intervention."
**Blinding of outcome assessment**	High risk	Quote: "The self-report psychosocial measures were collected at baseline, and at 3-, 6-, and 9-month follow-ups using electronic questionnaires on a tablet". Comment: psychological outcomes are self-reported by participants who were not blinded to intervention; therefore, outcomes assessment is not blinded.
**Incomplete outcome data**	High risk	Comment: 17/150 (11.3%) in intervention group, 23/150 (15.3%) in control group did not complete follow up for reasons (refuse to continue or did not have time) probably related to treatments.
**Selecting reporting**	Low risk	All outcome domains mentioned in protocol are assessed in the report.
**Other bias**	Some concerns	Concurrent treatment participants might receive is not documented and contamination of interventions were not mentioned.

Jones (2016)

Bias	Judgement	Support for judgement
**Random sequence generation**	Some concerns	Quote: "Participants were enrolled and randomized to condition as follows".<br><br>Comment: The term "randomized" is mentioned, no further information to support the judgement.
**Allocation concealment**	Some concerns	No information provided.
**Blinding of participants and personnel**	Some concerns	No information provided.
**Blinding of outcome assessment**	Some concerns	No information provided.
**Incomplete outcome data**	Low risk	The original sample size was 120 and the number of participants included in analysis is also 120. Comment: No loss to follow up.
**Selecting reporting**	Low risk	Expected outcomes were reported in this article.
**Other bias**	Some concerns	Concurrent treatment participants might receive is not documented and contamination of interventions were not mentioned.

Li (2017)

Bias	Judgement	Support for judgement
**Random sequence generation**	Some concerns	Quote: “the two groups of villages in each county were randomized to either a control condition or an intervention condition”. Comment: The term “randomized” is mentioned, no further information to support the judgement.
**Allocation concealment**	Some concerns	No information provided.
**Blinding of participants and personnel**	Some concerns	Method of blinding is not mentioned
**Blinding of outcome assessment**	Some concerns	Method of blinding is not mentioned
**Incomplete outcome data**	High risk	Comment: 24/257 (9.3%) in intervention group, 43/265 (16.2%) in control group did not complete follow up. Distribution of attrition between groups is different.
**Selecting reporting**	High risk	Mean differences of depression outcomes between groups are reported; however, mean depression scores at 6, 12, 18 and 24 month are not reported. Insufficient information of depression results for meta-analysis
**Other bias**	High risk	Comment: incorrect analysis due to lack of consideration of clustering.<br><br>Information of concurrent treatments individuals receive is unknown.

Li (2021)

Bias	Judgement	Support for judgement
**Random sequence generation**	Low risk	Quote: “Block randomization (size = 6) was used, and randomization was performed using a computerized random number generator.”
**Allocation concealment**	Low risk	Quote: “Allocation was concealed from all participants and field workers by using sealed and sequentially numbered envelopes.”
**Blinding of participants and personnel**	Some concerns	Method of blinding is not mentioned.
**Blinding of outcome assessment**	Some concerns	Method of blinding is not mentioned.
**Incomplete outcome data**	High risk	Comment: 17/129 (13.2%) in Three Good Things (TGT) with electronic social networking, 19/139 (13.7%) in TGT-only group, 19/136 (14.0%) in control group did not complete follow up. High attrition rate.
**Selecting reporting**	Low risk	Expected outcomes were reported in this article.
**Other bias**	High risk	Quote: “The baseline differences in background characteristics (i.e. socio-demographic characteristics, HIV-related characteristics and social-psychological outcomes) across the three groups were not statistically different; 24.2% of the participants in the TGT-SN group have told the exercise to other HIVMSM who also participated in the study, which may partially explain the contamination of the control group;(2.2%) participated in other psychological counselling or interventions during the intervention period (representing potential external contamination)”. Comment: Potential internal and external contamination.

Meffert (2021)

Bias	Judgement	Support for judgement
**Random sequence generation**	Low risk	Quote: "The study coordinator generated a random sequence of 2 colors in Excel corresponding to IPT+TAU and Wait List+TAU in blocks of 10."
**Allocation concealment**	Low risk	Quote: "The study coordinator generated a random sequence of 2 colors in Excel corresponding to IPT+TAU and Wait List+TAU in blocks of 10."
**Blinding of participants and personnel**	High risk	Quote: "The study coordinator and IPT therapists were not blind to group assignment."
**Blinding of outcome assessment**	Low risk	Quote: "Staff who gathered baseline and subsequent assessment data were blinded to group assignment."
**Incomplete outcome data**	High risk	Comment: 34/123 (27.6%) in intervention group, 16/133 (12.0%) in control group did not complete follow up. High attrition rate.
**Selecting reporting**	Low risk	All outcome domains mentioned in protocol are assessed in the report.
**Other bias**	Some concerns	Concurrent treatment participants might receive is not documented and contamination of interventions were not mentioned.

Nakimuli-Mpungu (2020)

Bias	Judgement	Support for judgement
**Random sequence generation**	Some concerns	No information provided.
**Allocation concealment**	Low risk	Quote: "Randomization of health centers was achieved by urn (health center managers separately picked a paper containing the intervention allocation from a basket; ratio 1:1)".
**Blinding of participants and personnel**	High risk	Quote: "both experimental and control interventions were identifiable to participants and outcome assessors".
**Blinding of outcome assessment**	High risk	Quote: "By design, both experimental and control interventions were identifiable to participants and outcome assessors".
**Incomplete outcome data**	High risk	Comment: 55/578 (9.5%) in intervention group, 111/562 (19.8%) in control group did not complete follow up. Distribution of attrition between groups is different.
**Selecting reporting**	High risk	Outcomes of disability days and poverty index scores listed in protocol are not reported in published report. Depression scores measured by SRQ-20 were not available. Adjusted odds ratio of depression disorders at 12 month is not available.
**Other bias**	Some concerns	Comment: Individuals are naïve to antidepressants. However, concurrent treatment participants might receive is not documented.

Nyamathi (2012)

**Bias**	**Judgement**	**Support for judgement**
**Random sequence generation**	Some concerns	Quote: "Between these two, one was randomly selected to engage the intervention group, while the second engaged the UC group". Comment: The term "randomized" is mentioned, no further information to support the judgement.
**Allocation concealment**	Some concerns	No information provided.
**Blinding of participants and personnel**	Some concerns	Method of blinding is not mentioned.
**Blinding of outcome assessment**	Some concerns	Method of blinding is not mentioned.
**Incomplete outcome data**	Low risk	Quote: "All 68 women completed the six-month follow-up".
**Selecting reporting**	High risk	Knowledge about HIV mentioned in the method section was not reported in the result section.
**Other bias**	High risk	Comment: incorrect analysis due to lack of consideration of clustering and small sample size.

Petlzer (2012)

Bias	Judgement	Support for judgement
**Random sequence generation**	Low risk	Quote: “Participants were randomized into study condition using a table of random numbers following their baseline assessment”.
**Allocation concealment**	Some concerns	No information provided.
**Blinding of participants and personnel**	Some concerns	Method of blinding is not mentioned.
**Blinding of outcome assessment**	Some concerns	Method of blinding is not mentioned.
**Incomplete outcome data**	Low risk	Comment: 3/76 (3.9%) in intervention group, 2/76 (2.6%) in control group did not complete follow up. Low attrition rate.
**Selecting reporting**	Low risk	No selective reporting is found when compare method section with results section.
**Other bias**	Some concerns	Concurrent treatment participants might receive is not documented and contamination of interventions were not mentioned.

Petersen (2014)

Bias	Judgement	Support for judgement
**Random sequence generation**	Low risk	Quote: “participants were allocated to an intervention and control arm using computer generated random allocation by the third author, who had no knowledge of the participant scores”.
**Allocation concealment**	Low risk	Quote: “random allocation by the third author, who had no knowledge of the participant scores”.
**Blinding of participants and personnel**	Some concerns	Method of blinding is not mentioned.
**Blinding of outcome assessment**	Low risk	Quote: “The PHQ-9, HSCL-25 and MSPSS were administered to both the intervention and control cohorts by 3 independent fieldworkers who were not informed whether the participants were in the intervention or control arms”.
**Incomplete outcome data**	High risk	Quote: “Loss to follow up was high and the study was only able to follow-up 49% of the original sample at post evaluation assessment”.<br><br>Comment: 24/41 (58.5%) in intervention group, 18/35 (51.4%) in control group did not complete follow up. High attrition rate.
**Selecting reporting**	Low risk	No selective reporting is found when comparing method section with results section.
**Other bias**	High risk	Quote: “Chi-square (χ2) analysis did not reveal any significant differences in demographic characteristics between the intervention and control arms”. Comment: All characteristics are balanced at baseline. However, sample size was small,

Sarna (2008)

Bias	Judgement	Support for judgement
**Random sequence generation**	Low risk	Quote: "Computer-generated random number assignment was used, allocating an equal number of participants to treatment and control groups".
**Allocation concealment**	Low risk	Quote: "Allocation concealment was maintained with sequentially numbered, opaque sealed envelopes".
**Blinding of participants and personnel**	High risk	Quote: "Only laboratory personnel were blinded to study group allocation".<br><br>Comment: participants were not blinded.
**Blinding of outcome assessment**	Some concerns	Method of blinding is not mentioned.
**Incomplete outcome data**	High risk	Comment: 27/116 (23.3%) in intervention group, 24/118 (20.3%) in control group did not complete follow up. High attrition rate.
**Selecting reporting**	High risk	Depression symptoms mean scores at baseline and post-intervention are not reported. Only medium scores of differences between baseline and postintervention and p value between groups are displayed.
**Other bias**	Some concerns	Quote: "The m-DOTand control groups had similar baseline characteristics (Table 1)." Comment: All characteristics are balanced at baseline.<br><br>Concurrent treatment participants might receive is not documented.

Wang (2010)

Bias	Judgement	Support for judgement
**Random sequence generation**	Some concerns	Quote: "Randomized trial and pre-tests and post intervention tests were used in this study". Comment: The term "randomized" is mentioned, no further information to support the judgement.
**Allocation concealment**	Some concerns	No information provided.
**Blinding of participants and personnel**	Some concerns	Method of blinding is not mentioned.
**Blinding of outcome assessment**	Some concerns	Method of blinding is not mentioned.
**Incomplete outcome data**	High risk	Comment: 8/58 (13.8%) in intervention group, 10/58 (17.2%) in control group did not complete follow up. High attrition rate.
**Selecting reporting**	Low risk	No selective reporting is found when comparing method section with results section.
**Other bias**	Some concerns	Concurrent treatment participants might receive is not documented.

Williams (2014)

Bias	Judgement	Support for judgement
**Random sequence generation**	Low risk	Quote: “A stratified randomization procedure with a block size of 10 was used. A table was constructed using SAS programming to distribute intervention and control assignments in random order”.
**Allocation concealment**	Low risk	Quote: “A table was constructed using SAS programming to distribute intervention and control assignments in random order”.
**Blinding of participants and personnel**	Some concerns	Method of blinding is not mentioned.
**Blinding of outcome assessment**	Some concerns	Quote: “Trained interviewers collected data during structured face to-face interviews conducted in a private area at the time of a regularly scheduled monthly visit to the China CARES site.”<br><br>Comment: method of blinding is not stated.
**Incomplete outcome data**	High risk	3/55 (5.4%) in intervention group, 13/55 (23.6%) in control group did not complete follow up. High attrition rate. Distribution of attrition between groups is different.
**Selecting reporting**	Low risk	No selective reporting is found when comparing method section with results section.
**Other bias**	Some concerns	Social support is significant different between groups at baseline; however, it is adjusted for in multivariate analyses.<br><br>Concurrent treatment participants might receive is not documented.
